# A Middle Devonian vernal pool ecosystem provides a snapshot of the earliest forests

**DOI:** 10.1371/journal.pone.0255565

**Published:** 2021-09-01

**Authors:** Khudadad

**Affiliations:** Department of Biological Sciences, Binghamton University—State University of New York, Binghamton, New York, United States of America; Peking University, CHINA

## Abstract

The dichotomy of the earliest ecosystems into deltaic and floodplain forests was a long-standing view in paleobotany. The morphological traits such as nonbranching rootlets, bifurcating rhizomes, and bulbous bases of the primitive trees such as *Eospermatopteris* and lycopsids were considered adaptations to the lowland deltaic environments. In contrast, the traits of *Archaeopteris* trees such as wood, hierarchical branching networks of roots, and true leaves are an adaptation to the upland floodplain environments. The discovery of the Town of Cairo Highway Department (TCHD) fossil site in Upstate New York, where all major clades occupied a floodplain environment casts doubt on the validity of the environmental partition by the earliest trees at the higher taxonomic levels. This study aims to test the hypothesis of the environmental partition at the local scale by reconstructing the fossilized forest-floor landscape, the changes in the landscape over time, and the distribution patterns of the trees along the local environmental gradient at the TCHD site. To reconstruct the fossilized forest floor and to determine the environmental variations at the local scale, seven parallel cross-sections were drawn from south to north at THCH. The outcrop at the quarry floor measured 300 m in the north-south, and varied around 100–150 m in east-west direction. Primary sedimentary structures, the thickness of the sedimentary deposits that formed the forest floor and the surrounding quarry walls, paleosol features, and mega-fossils were measured, recorded, described, and mapped. Up to 3 meters deep drill-cores were extracted from the forest floor. The data was used to correlate the sedimentary deposits, and reconstruct the preserved landscape. Three dominant landscape features including an abandoned channel, an old-grown forest, and a local depression were recognized. These landscape features influenced greatly the pattern of local drainage, slope-gradients, patterns and durations of seasonal water pooling, paleosol developments, fossil distribution, and depositional environments. There is no evidence of environmental partitioning by trees at higher taxonomic levels at the local scale. The size and morphology of the root systems didn’t determine the distribution of the trees along the local environmental gradient such as drainage patterns but played important roles in tree stabilization. Forests would go through self-thinning as they matured. Upon comparison, it was found that the forests in unstable environments showed greater resiliency compared to forests established in the stable environments.

## Introduction

Arborescence evolved independently in different earliest tree clades, e.g., lycopsids, fern-like cladoxylopsids, and progymnosperms. While these trees shared common upright growth of the central trunk supporting a crown of branches, they differed in their vascular systems, mechanical support tissues, leaves or leaf-like photosynthetic organs, and root morphologies [1]. Anerophytalean progymnosperms were an exception, as despite being among the earliest tree-sized plants that evolved wood in the construction of their vasculature and mechanical tissues, they showed rhizomatic stems that ran between trees and possibly climbed onto them [2]. Because of the rhizomatic stems, the aneurophytales could not be strictly defined as trees, but with stem diameters comparable to young trees, possibly tree-sized heights, and being part of the earliest forest ecosystems, the evolution of the aenurophytalean body plans could be considered as a unique mode of arborescence. An accepted model for the spatial distributions of the earliest forests was based on environmental specialization: the trees with simple root morphologies -non-branching adventitious rootlets, and likewise bifurcating rhizomatic roots system- adapted to the deltaic wetlands, and the trees with near-modern root systems -branched root networks (BN)- occupied riparian environments [3–5]. The partition of lowland environments by the plants at the higher taxonomic levels was also suggested for the Late Devonian forests [6]. These kinds of ecosystems were only reported from the Paleozoic era.

The fossilized forests of the cladoxylopsid trees at the Riverside quarry Gilboa were considered as evidence of the Earth’s oldest forest [7]. The fossils were recovered in the form of stump-casts that had bulbous bases and also in the form of the stump footprints consisting of central depressions formed by the bulbous bases of the tree. The central depressions were surrounded by mounds of shale-mud and fossilized rootlets. Goldring interpreted the sediments that hosted the Gilboa stumps as shale-mud formed in the swamp environment of the vast delta of the Devonian shoreline, and the bulbous bases of the trees as an adaptive morphology to the deltaic environments [7]. The stump-casts were found in coarse sand at three horizons and interpreted as forest regeneration after quick burial in an unstable deltaic environment. Goldring also reported variations in the stumps shapes and the mechanical supporting tissues, and divided the stumps into Textilis and Erianus-type stumps. Textilis-type stumps had rapid narrowing -tapering- above the bulbous base. A network of the interlacing strands that covered the bulbous base and trunks of the trees provided mechanical support. In contrast, Erianus-type stumps gradually narrowed above the base and had parallel strands for mechanical support. The stumps were considered to be a seed-fern, and named *Eospermatopteris* [7]. Recent studies added greatly to our understanding of the *Eospermatopteris* basic body plan. The trees grew upright, could grow more than 8 meters tall [2], and had a crown of branches [8] that lacked leaves. The trunk had a large central pith mostly composed of the parenchyma tissue [9] and the vascular tissues were limited to the perimeter of the trunk. The adventitious non-branching rootlets added additional layers of mechanical support to the stems, and by penetrating the paleosols anchored the trees to the ground. Some recent workers confirmed Goldring’s interpretation that the *Eospermatopteris* trees adapted to the deltaic environments. Mintz observed that the *Eospermatopteris* forest at Gilboa was established on fine-grained sandstone of deltaic swamps, where small tree communities had clustered distribution while large tree communities had random distributions [4]. Stein mapped 1200 m^2^ of the Riverside Quarry at Gilboa and observed limited root penetration by the *Eospermatopteris* rootlets in the dark gray sandy mudstone which was interpreted as a wetland of coastal plain environment [2]. Retallack suggested that cladoxylopsid trees such as *Eospermatopeteris* were mangals that tolerated 7°C less than the current limits of the modern mangroves [5] as New York and Pennsylvania located at the paleolatitude 35°S.

Arborescent lycopsids dominated lower and Middle Pennsylvanian swamp environments [10], while the oldest records of this group were reported from the Portage beds of central New York [7] and the Riverside Quarry at Gilboa [2]. The presence of the arborescent lycopsids in the oldest forests was evidence for the convergent evolution of arborescence in different plant clades, and the evidence from deltaic deposits at Gilboa showed the adaptation of this group to waterlogged environments. Goldring didn’t provide details of the arborescent lycopsid that she mentioned. Likewise, only a part of the trunk and the branch axes of a lycopsid tree were preserved at the Riverside Quarry at Gilboa [2]. The dimensions of the preserved materials such as 3.9 meters of length, and tapering from 15 to 11 cm diameters of the stem were taken as evidence for arborescence. The most informative features of the arborescent lycopsids that have been used for the identifications include leaf cushions -leaf base scars- on stem and branch axes, dichotomous branches of the crown, and likewise the bifurcating root systems. Determinate growth of branch axes of the tree crowns were another distinctive feature of the arborescent lycopsids [10].

Stump and root cast interpreted as archaeopterid progymnosperm were reported from the Plattekill Formation at West Saugerties locality of Upstate New York [4]. The poorly developed paleosols that hosted the trees formed in the floodplain environment. Based on his studies of multiple localities, Mintz concluded that *Eospermatopteris* occupied deltaic environments and archaeopterids occupied floodplain environments [4]. Using proxies from paleosols, Retallack supported Mintz’s conclusion that archaeopterids formed riparian woodlands and cladoxylopsid trees deltaic mangals [5]. He proposed that progymnosperms trees were more limited by precipitation than temperature, and migrated from high latitudes or altitudes during early Givetian when the climate was warmer and wetter. Likewise, the large cladoxylopsid trees migrated from wetter-warmer regions during warm-wet climatic conditions and appeared in sulfidic intertidal paleosols and formed mangal forests. The evolutionary innovations that resulted in size and architectural variations during vascular plant evolution from herbaceous to tree-size plants were considered to be the cause of the partitioning of lowland and highland environments at higher taxonomic levels [3].

Stein reported [11] a Mid-Givetian well preserved forest floor from the TCHD that was 2–3 million years older than the Gilboa forests. The site hosted tree roots belonging to the *Eospermatopteris* similar to the famous Gilboa tree types. In contrast to the deltaic environment of the Gilboa, the TCHD experienced periodic drier conditions. Additionally, well-preserved root systems that belonged to the oldest known rhizomatic lycopsid and likewise conifer-like archaeopterids were found at the site. Up to 3 meters drill cores were extracted from the paleosol in the proximity of the root systems which showed two stacks of well-developed paleosols. The overlying paleosol was 1.6 meters deep and root traces were observed across the depth of the soils [12]. As the root depths in paleosol have been used as a proxy for water table depth [5], the core-extracts from the TCHD indicated the water table fluctuated to several meters. It was likely that such water table fluctuations were challenging for trees like *Eospermatopteris* that had non-branching and shallow roots. The presence of earliest trees belonging to three tree clades in a drier environment exposed the inadequacies of the previous models that have suggested morphological adaptations to the mangal and riparian woodland [5], clade-level segregation of forests into *Eospermatopteris*-forest of deltaic-swamps, and *Archaeopteris-*forests of fluvial-floodplains [4], and furthermore, progressive colonization of the land environments from wet lowland to drier highland as the vascular plants sizes increased as a result of evolutionary innovations [3]. The presence of cladoxylopsids and lycopsid trees in both the wet environment of the Gilboa, and the drier environment of the TCHD asks for re-evaluations of morphological features such as bulbous bases, adventitious rootlets, and rhizomatic root habits, particularly their interactions with the paleosols that experienced environmental stresses such as seasonal drought. Similarly, the absence of *Archaeopteris* trees -the earliest known trees with wood anatomy and growth habits like modern trees [13]- in deltaic environments needs to be explained. Furthermore, the presence of trees that varied greatly in their morphologies in the same depositional environment demonstrated the possible importance of the local environmental variability in determining the spatial distribution of the trees.

The new evidence for the complex earliest forests point to the possibility of complex ecosystem processes. Similarly the variations in forest compositions between the fossil localities that are geographically close suggests the possible roles of local processes in shaping the compositions of the forests. Here we list several questions that we consider important to explore in order to understand the greening of the Earth in the Devonian Period. (a) Devonian was a period when the rate of phenotypic innovations were high among vascular plants [14] but there was a discrepancy of phenotypic innovations between above and belowground parts of the plants. Most of the innovations occurred in aboveground parts of plants which indicated the high selective pressures on the aboveground parts of plants. The discrepancy of phenotypic variations between different plant parts poses a question on the role of environmental stability in establishment of the earliest forests, and the subsequent formation of complex forests. (b) While tree-like growth habits had obvious advantages in terms of light-harvesting and spore dispersal, tree-like plants were also more prone to extinction when environments changed rapidly than herbaceous plants [14]. The independent evolution of morphological and anatomical complexity in different vascular plant clades suggested long stable environments that allowed the evolution of tree-like plants. Since the tree clades weren’t localized phenomena, and had global distributions [15], it was very likely that these early tree clades experienced periods of unstable environments, and it would be interesting to learn how these tree clades fared in variable environments, and explore the possible environmental constraints in each major tree clades. (c) It has been suggested that the trees with deeper roots such as *Archaeopteris* increased the silicate rock weathering and stabilized lands [3]. It needs to be investigated to determine if the *Archaeopteris* trees increased the stability of the landscapes or they preferred the environments with stable landscapes? (d) It has been indicated that arborescent lycopsids [16] and *Eospermatopteris* trees [17] had high growth rates and short lifespans. In comparison, woody *Archaeopteris* trees had indeterminate growth and lived long lives. Since all the tree-clades were free-sporing and very likely the gametophytes of such trees did not have protection against desiccation [15], it would be interesting to explore the interactions of the tree-clades with drainage patterns of the forested landscapes and if possible the seasonal patterns of the drainage patterns. (e) It has been proposed that the evolution of vascular plants with root systems diversified on the Paleozoic landscapes by introducing meandering channels and muddy floodplains by stabilizing the river banks [18]. It would be interesting to explore the modification of the landscapes at the local levels, as tree roots redistributed water in the paleosols by drawing water from the ground. By drawing water during wet seasons, the trees could have influenced the rate of clay expansion, and in the dry season, the shades from tree canopies and water redistributions by roots could have affected the sizes of the desiccation cracks, as well as developments of the slickenside.

This study aims to use the paleosol features and primary sedimentary structures at the TCHD to reconstruct the Middle Devonian landscape that hosted the mixed forest at this site, determine the local environmental variability such as dominant drainage patterns, and investigate its possible influence in the spatial distribution of the trees based on the tree clades and sizes. This study will also attempt to compare the findings from TCHD to the other Middle Devonian fossilized forests in order to understand the ecosystem processes that resulted in the partitioning of the land environments at the higher taxonomic levels as was suggested previously.

## Study site and methods

**Fig 1:**
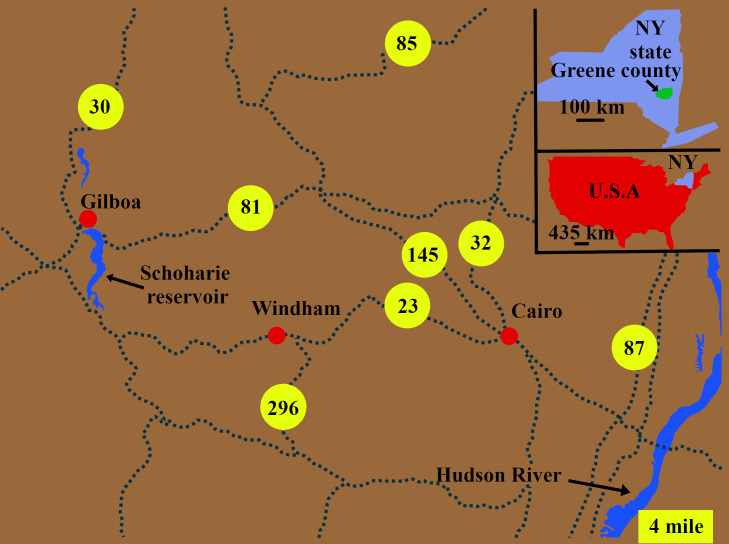
The study site is located at the Town of Cairo Highway Department quarry. The study site is also known as the Town of Cairo Highway Department Fossil Site (TCHD). The Town of Cairo is located along the routes NY-23 and NY-32 at the west of the Hudson River in Greene County, New York State, USA.

The Appalachian orogeny was one of the important mountain-building events during the Paleozoic era that formed the Appalachian foreland basin and Catskill river systems. The Appalachian basin was positioned 5°-20° south of the equator and had a semi-arid climate [5]. The tephra from the volcanic activity of the island arcs in the orogenic subduction zones provided the parent material for the smectite clays which were responsible for the developments of the frequent paleo-vertisols in the Catskill region [19]. The succession of sandstone and mudstone deposited by the river systems in the up-valley and deltaic regions of the basin have been preserved in the form of the Middle Plattekill and Manorkill Formations of New York State. The single-channels of the river system in the coastal areas were tens of meters wide, and with increasing distance from the coast, the river became wider that reached to the hundreds of meters in width [20]. The Appalachian basin hosted the oldest forests so far known. The fossilized forest floor, paleo-vertisol, and associated sediments explored in this study were deposited in the Middle Devonian by the Catskill river system in the Appalachian foreland basin. The fossil site is located at the TCHD, Greene County, New York State, USA (Fig 1). The town of Cairo is located about 35 miles south of Albany and ten miles west of the Hudson River. TCHD is located between routes NY-145 and NY-23, nearly 2 kilometers northeast of the Town of Cairo office. The roadside quarry is owned and operated by the Town of Cairo highway department.

**Fig 2:**
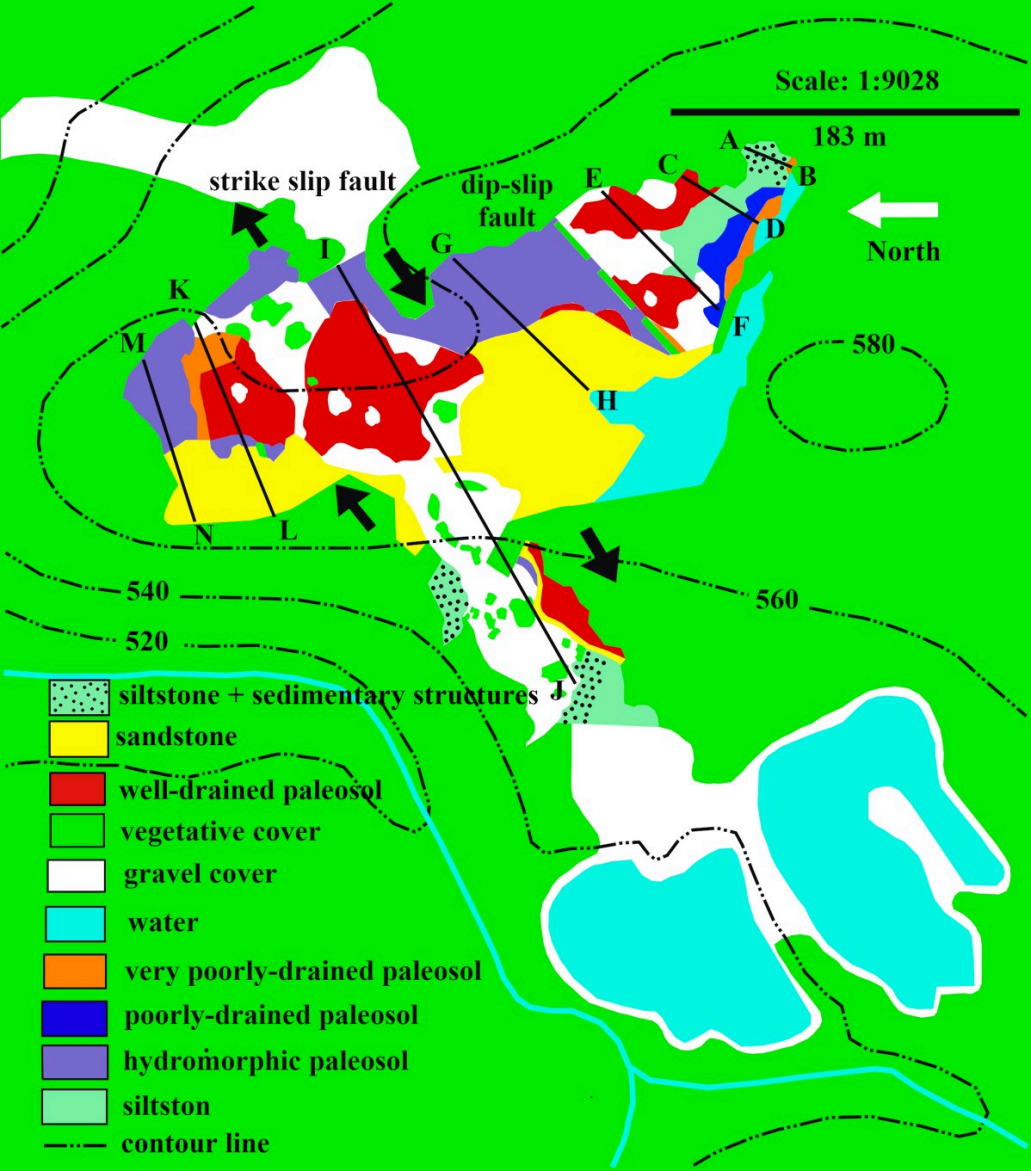
An aerial map of the sediment exposure at the TCHD. The aerial exposure map of the sediment exposures were drawn on a USGS base map. The planar exposure of the sediment deposits and covered areas are represented by different colors. Parallel cross-sections on the map were named alphabetically from south to the north of the quarry. The cross-section A-B located at the east-southeast, and the M-N at the north-northwest of the quarry floor. The Dip-slip fault was marked with a dashed line and the strike-slip fault with arrows.

The quarry floor showed considerable variations in surface paleosol features, fossils, and the distributions of the capping sediments. An aerial map of the sediment exposures at the TCHD was drawn on the USGS base map based on the field observations. Eight parallel sedimentological cross-sections were drawn on the aerial map of the TCHD (Fig 2). The cross-sections were named in ascending alphabetical pairs, such as A-B, C-D, and so on from south to the north of the quarry floor. The cross-section profiles aimed to reconstruct the fossilized landscape by following the changes in the thickness of sediment deposits, particle sizes, sedimentary structures, as well as surface paleosol features formed as a result of variations in the local topography, drainage patterns, and bioturbation.

**Fig 3:**
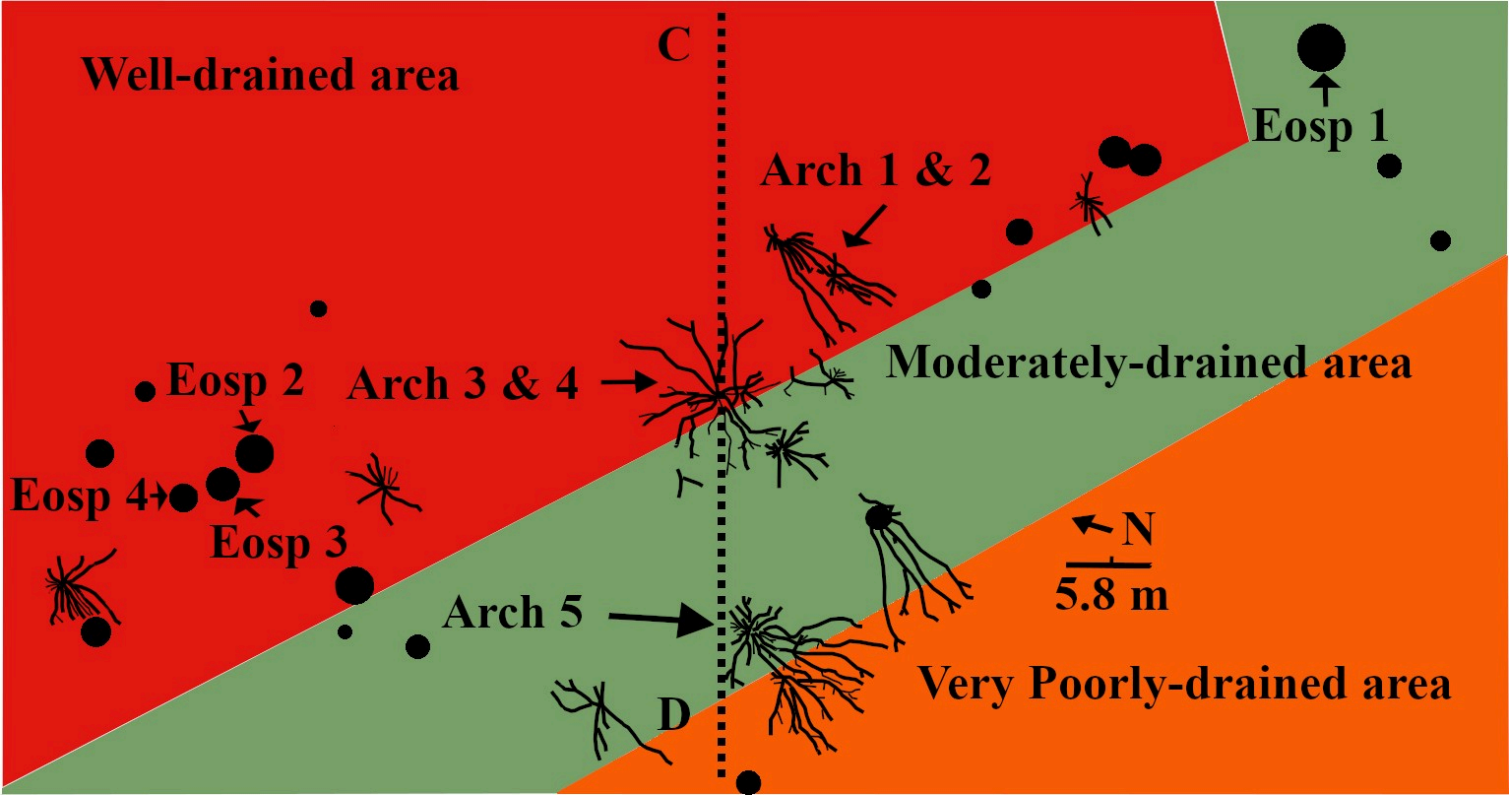
The distribution of the fossilized root systems belonging to three different tree clades. The tree distributions at the TCHD were mapped using Stein’s map as base-map [11]. Stein’s map only covered the exposed fossils around the C-D cross-section (Fig 2). Arch stands for *Archaeopteris*, Eosp for *Eospermatopteris*, and Lyc Tree for the lycopsid tree. Note L- shaped distributions of the Eosp trees in the form of the two clusters (one around Eosp 1, and another around Eosp 2). Likewise, note the distribution of the Arch tree from Arch 1 to Arch-5. The linear distribution extends to Arch 6 which is not shown in this map. The colors in the map represent the dominant drainage patterns in the fossilized landscape that were inferred from the redox coloration. The actual surface paleosol colors were light-red, dark-gray, and yellowish-brown indicating the existence of a drainage gradient from well-drained, moderately drained to poorly drained area.

**Fig 4:**
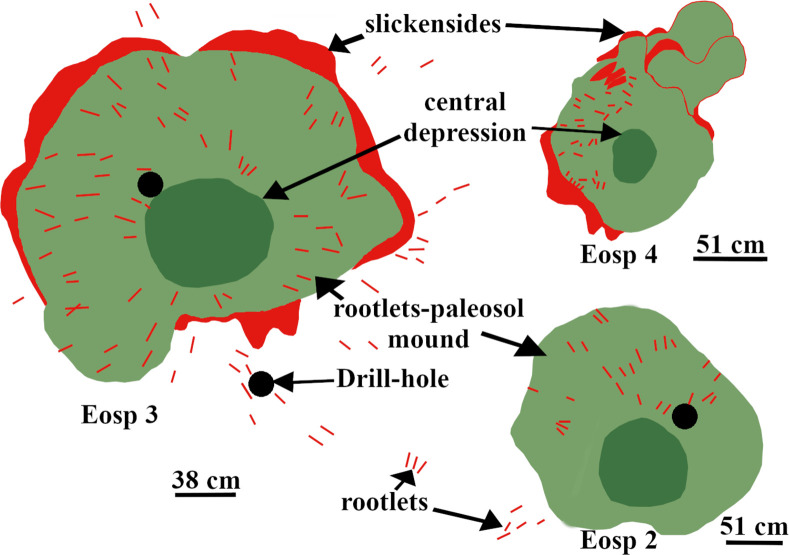
The main features of the *Eospermatopteris* 2, 3 and 4. The main features of the *Eospermatopteris* root-mold included a central depression, rootlets-paleosol mound that surround the central depression, rootlets, and slickensides. The central depression formed by the bulbous bases of the *Eospermatopteris* trees. The rootlets-paleosol mound formed by the mantle of rootlets that emanated from the bulbous bases and hugged the paleosol. Slickensides developed by the differential vertic movements as a result of seasonal swell-shrink of the paleosols, and the rootlets-paleosol mound. Note that the Eosp 3 shows multiple concentric slickensides, Eosp 2 strong slickenside at the border between the rootlets-paleosol mound and the host paleosol, and no visible slickensides observable at the boundary of Eosp 4. The bulbous base and the rootlets-paleosol mound acted as a single unit to stabilize trees.

**Fig 5:**
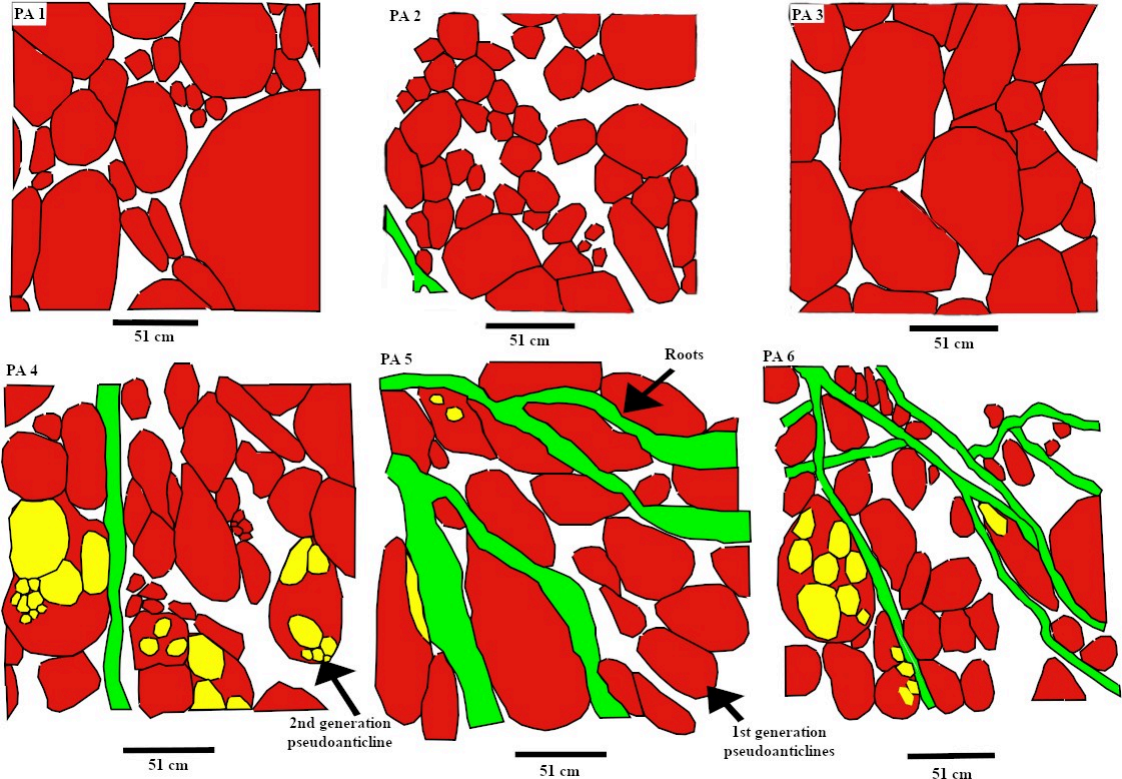
The variation in the sizes of the pseudoanticlines in rooted and unrooted areas. Roots trace fossils are shown in green color, the first generation pseudoanticlines in red, and the second generation in yellow colors. PA 1–3 is the maps of the pseudoanticlines in the unrooted areas along section C-D. The PA-4 is the map of the pseudoanticlines in the proximal area of the lycopsid root system. PA-5 is the map of the pseudoanticlines in the proximal area, and PA-6 in the distal area of Arch-5.

The spatial distributions of the in-situ root systems belonging to three tree clades were marked and named (Fig 3, see S1 and S2 Tables via https://doi.org/10.6084/m9.figshare.14556270.v4) on a map. Stein’s map [11] was used as a base-map for consistency with previous studies at the TCHD. The fossilized roots of *Archaeopteris* trees were named Arch-1, Arch-2, and so forth along the C-D section. *Eospermatopteris* root-base molds and the associated rootlet-paleosol mounds were plan-mapped on the scale of 5*5 inches, and named as Eosp-1, Eosp-2, and so forth (Fig 4, see S2 Table via https://doi.org/10.6084/m9.figshare.14556270.v4). In order to record the interactions of root-soil and root-drainage patterns, the paleosol surface undulations (pseudoanticlines) from proximal, distal part of root systems, as well as non-rooting areas were plan-mapped on 5*5 inches grids. The mapped pseudoanticlines were named PA-1, PA-2, and so forth (Fig 5, see S4 Table via https://doi.org/10.6084/m9.figshare.14556270.v4). This study defines pseudoanticlines as hardened paleosol surface undulations that are composed of microhighs and microlows that formed as a result of repeated wetting and drying. As proximal roots are the earliest roots that develop, this study uses the interactions of the proximal roots with pseudoanticlines as proxy for stability of the surface soils (non-deposition) during root developments. As macro-scale slickenside (decimeter diameters) indicates churning of soil that has not yet developed a hardened surface-soil, hence slickensides are taken as proxies for young paleosol. The term macro-scale slickenside is used to distinguish the regular slickensides that are main features of the vertic paleosols from the centimeter scale slickensides that developed around roots. In similar fashion, decimeter and centimeter paleosol features were categorized into two separate classes. For instance, the term pseudoanticlines is used for regular pseudoanticlines that are decimeters in diameter, and the term micro-pseudoanticline used for the centimeter-sized pseudoanticlines. By the similar token, the term jumbo-cracks were used to distinguish decimeter-sized desiccation cracks from centimeter-sized cracks.

**Fig 6:**
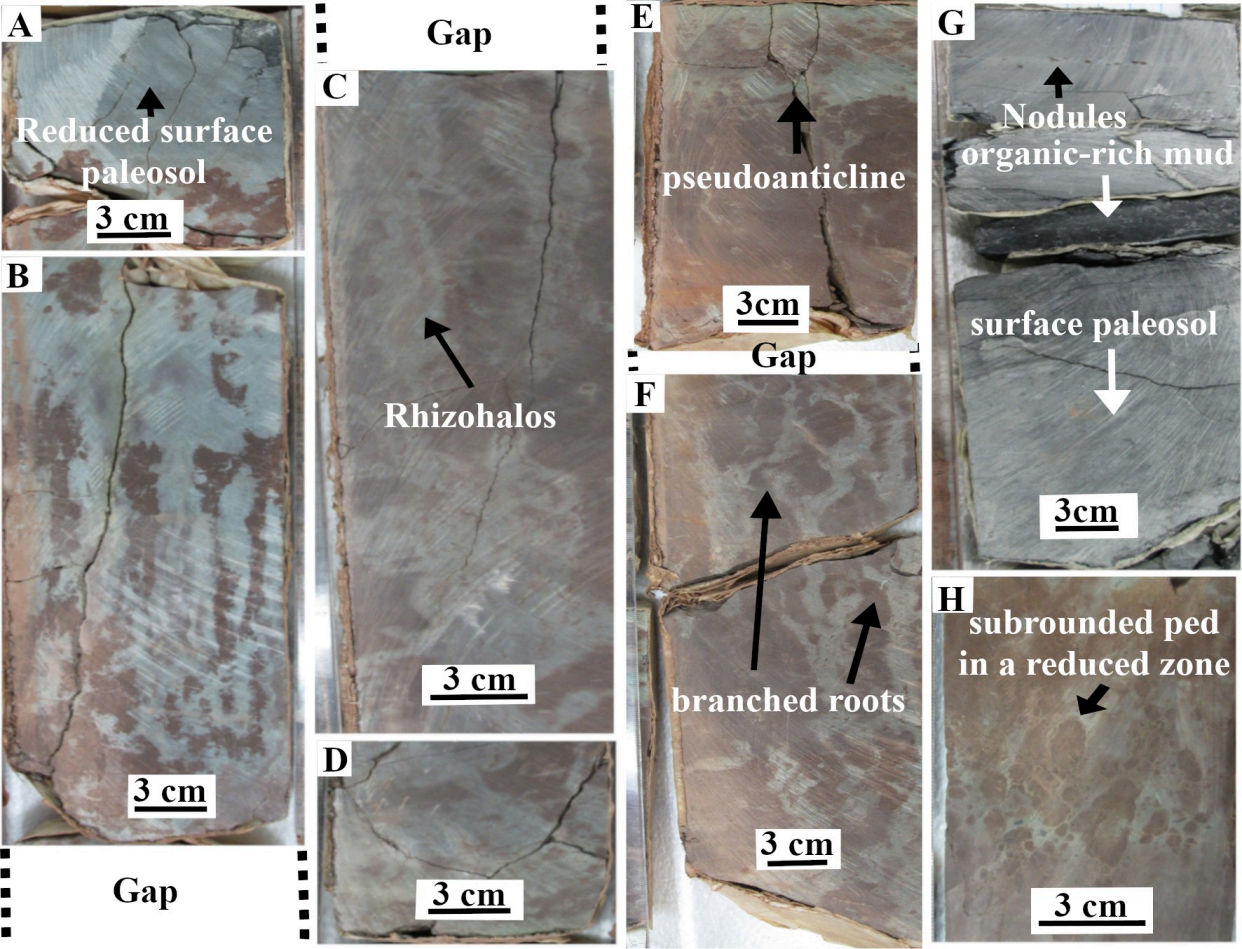
Drill-core extracts from the TCHD. The core-extracts were selected that possessed the representative features of the subsoil (Fig 6A–6F and 6H) from the well-drained areas as well as hydromorphic paleosol (Fig 6G). (A) Note the reduced surface paleosol in comparison to the more oxic subsoil. (B) Reduced areas are dominant around rhizohalos. The almost vertical rhizohalos indicated that roots didn’t face resistance in the deep soil. (C) Note the branching root-casts (marked by arrow) surrounded by the rhizohalos. (D) The figure shows the end of top-paleosol and the beginning of underlying paleosol. The underlying paleosol has a reduction-zone followed by a desiccation zone (E). (F) The figure shows the rooting zone of the underlying paleosol where the branched root system is observable. (G) A core extract from hydromorphic paleosol: Note the iron-manganese zone, organic-rich zone, and underlying surface hydromorphic paleosol recorded variations in moisture-saturation of the paleosol. (H) A desiccation zone from the well-drained paleo-vertisol: The orientation of the subrounded peds in the reduced matrix, and likewise, the subrounded organic materials are notable.

Drill cores extracted from paleosols near the *Eospermatopteris* and *Archaeopteris* root systems (Fig 6, see S1 Fig via https://doi.org/10.6084/m9.figshare.14558445.v1). The core extracts were initially reported by Morris [12]. The locations of the core extracts were recorded on Stein’s map [11] (see S5 Table via https://doi.org/10.6084/m9.figshare.14556270.v4), and named in numerical order. Furthermore, they were cut in halves, and kept in the New York State Museum at Albany (NYSM). The depths of the paleo-vertisols, root systems, desiccation cracks, and variations in redoximorphic features were additional information that core extracts provided. The depths of root systems were used as a proxy for the depths of the water table, and redoximorphic features for drainage patterns. In soils with shallow water tables, the roots tend to be shallow therefore the deeper fossilized roots have been used as a proxy for deeper water tables in the paleosols [5]. The redox coloration of paleosols as a result of changes in the paleosol-moisture is called redoximorphic features [21]. Redoximorphic features develop in saturated soils where oxygen is depleted, and bacterias reduce ferric (Fe^3+^) to ferrous iron (Fe^2+^), and likewise manganic (Mn^4+^) to manganese (Mn^2+^) forms. Areas of the soils where the iron is reduced develop bluish-gray and greenish-grey. When saturated soil gets exposed to the air, the dissolved forms of the iron get oxidized. The areas of soils with relatively higher concentration of the iron-oxides develop reddish colors. The chroma of the redoximorphic features is associated with the duration of the wetness [22]. In hydromorphic soils, the high chroma inside peds can occur in a very short period of a day or less of soil saturation. The soils that experience saturation for a few days [23] develop a chroma of two. The chroma values less than two are associated with the saturation of a few months [23]. Munsell’s color book was used to describe the color and chroma of the redoximorphic features.

As roots fossilized in different forms, we used the umbrella term rhizolith for all types of fossilized roots and used Kraus and Hasiotis nomenclature [21] to describe the modes of the rhizolith preservation including mold, cast, rhizohalos, carbonaceous roots, and impressions. Root-molds are trace fossils where only the general morphology of the roots have been preserved in the form of the voids in the paleosols. Cast-roots are root-voids filled with sediments. Rhizohalos are reduced or oxidized areas developed around the roots. Carbonaceous roots are body fossils that are preserved in the form of carbonaceous materials. The impressions fossils mostly belong to aboveground parts of the trees such as the stem where a carbonaceous film is preserved. The term rootlets were used specifically for the equidimensional roots of the *Eospermatopteris* and lycopsid trees. For *Archaeopteris* roots, the terms main-roots, proximal, distal, and fine roots were used. The roots that were directly attached to the trunk were called main-roots and millimeter-sized roots (1 to <2 mm) were categorized as fine roots. We used USDA’s field book for describing and sampling soils (1998) [24] to record and describe a number of the local paleosols features including, slope shapes, redoximorphic size classes, pedogenic (ped) structures, and sizes. The local slope was inferred based on the local drainage patterns. The size of the redoximorphic features were classified into fine (< 2mm), medium (2 to < 5mm), coarse (5 to < 20 mm), very coarse (20 to < 76 mm), and extremely coarse (> 76mm). The pedogenic structures were categorized as granular, blocky, platy, wedge, and prismatic peds. Small peds with irregular faces were called granular, the polyhedral peds with planar faces blocky-peds, the peds with platy-like units platy-ped, peds with interlocking lenses wedged-shaped peds, and vertically elongated peds prismatic-peds. The ped sizes were described as fine (< 5 mm in diameter), medium (5 to < 10 mm in diameter), medium (10 to < 20 mm in diameter), coarse (20 to < 50 mm in diameter) and very coarse (> 50 mm in diameter).

**Fig 7:**
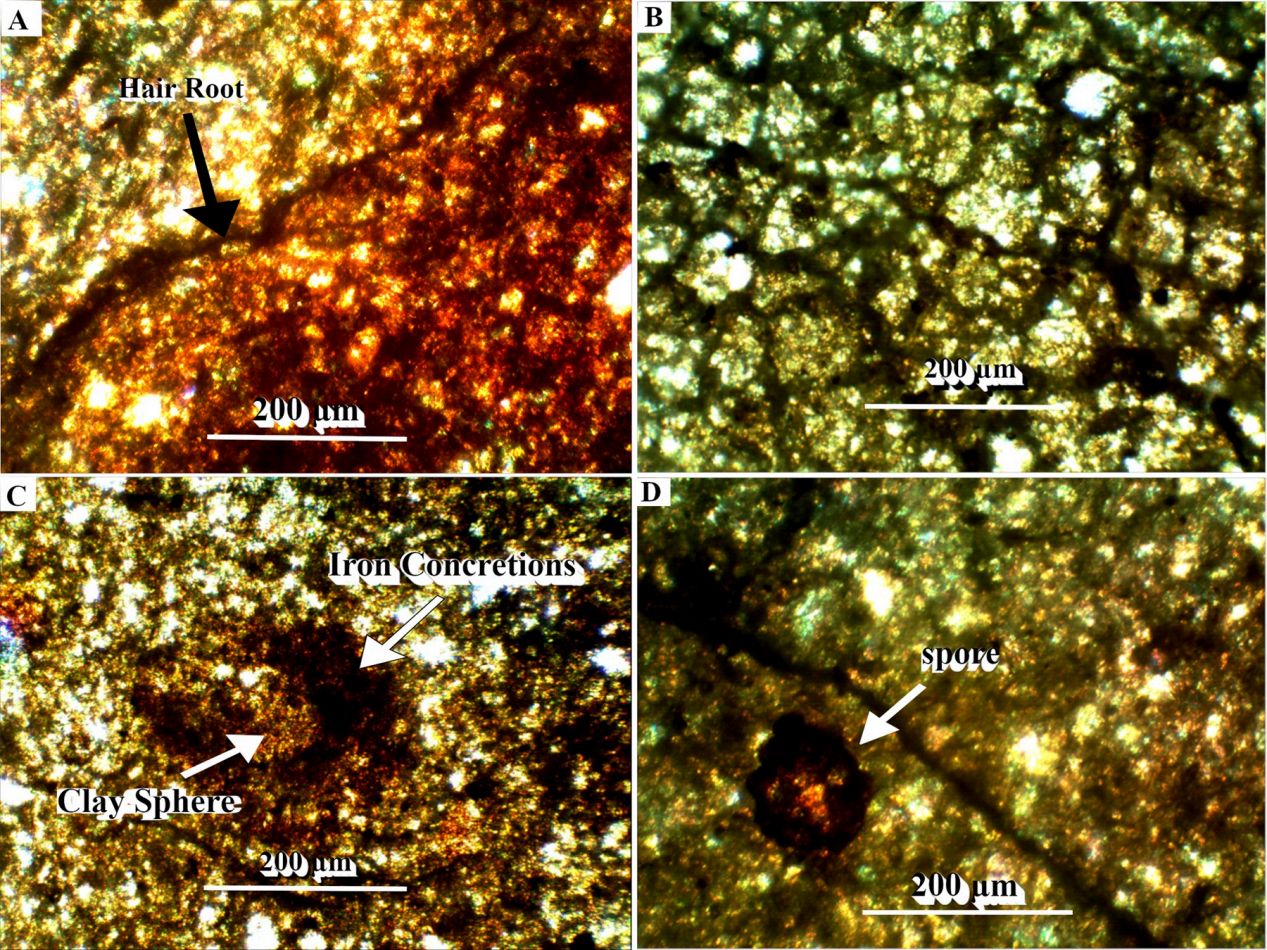
Photomicrograph from the fine root areas along the C-D section. (A) A fine root working as a borderline between oxidized and reduced sediments. (B). The network of dense fine roots. (C) An iron concretion formed around a spherical clay. (D) A Spore along a fine root.

**Fig 8:**
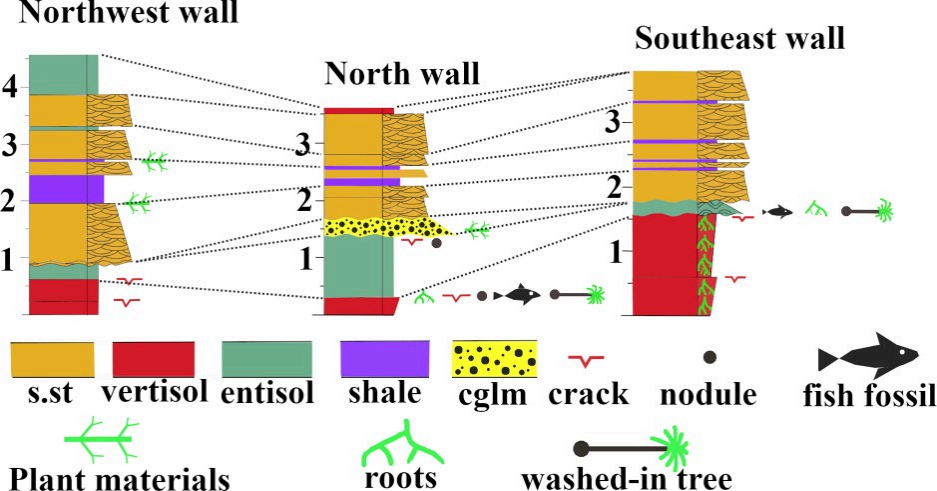
Correlations of the north, northwest, and southeast at the TCHD quarry-walls sediment deposits. The sediment deposits, paleosols, and fossils from the quarry walls, floor and the drill-cores were used to construct the sedimentary profiles. For instance, the information on the depth of the vertisols from the drill-core extracts were included in the profile of the southeast wall. The Northwest wall profile showed more variations as the strike-slip fault made a near 3-D exposure of the wall. Likewise, the quarry floor made it possible to trace the paleosols that made the forest floor as well as the capping sediments from south to north. The fossils included in-situ rooting as well as washed-in fossils such as fish and trees which were helpful in the correlations of the paleosols exposed on the quarry floor. The primary sedimentary structures and the tracing of the sedimentary deposits across the quarry walls made it possible to correlate the sedimentary deposits of the wall. There were four sequences of deposits from base to the top of walls including, trough cross-bedded sandstone, heterolithic bedding, followed by trough cross-bedded sandstone, and paleosols, respectively that varied in their thickness between the walls.

**Fig 9:**
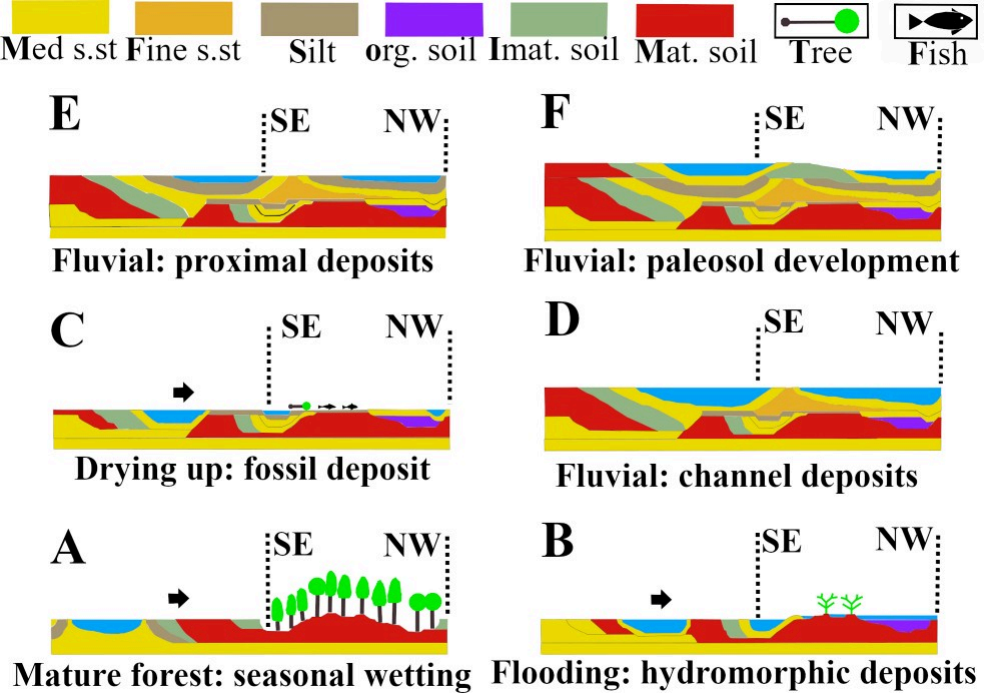
Interpretation of the depositional environments of the TCHD. The actual deposits of the TCHD are limited to the two vertical dashed lines shown in the figure. rooted area. The interpretation of the areas between the dashed line are extrapolations based on the thinning or thickening of the sediments at the TCHD: (A) A forest on an abandoned channel on a distant floodplain. (B) Crevasse splays changed the depositional environment from seasonal-pooling to permanent-pooling which resulted in the disappearance of the forest and preservation of the (C) A flood deposited washed-in fishes and young trees. (D) The crevasse splays and flood changed the local landscape by partially filling the local depression. Lateral migration of a local channel scoured part of the local depression and changed the depositional environment to an active channel. (E) Another lateral migration resulted in a channel bank deposit in the form of the heterolithic beds on the northwest side of the TCHD (F) Yet another episode of lateral migration followed by stabilization of the channel-bank at the northwest side of the TCHD that resulted in the development of the paleosols there.

**Fig 10:**
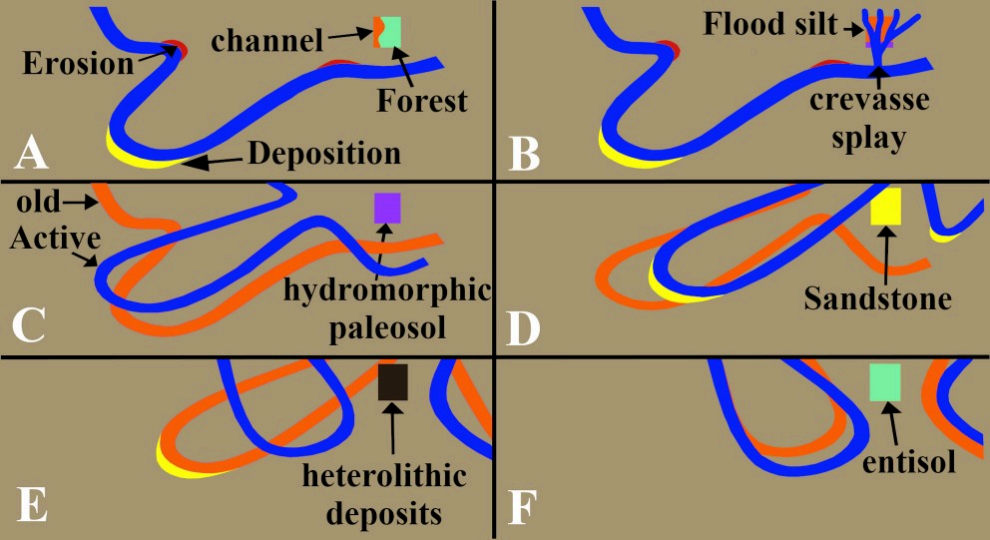
Interpretation of deposits associated with the lateral migration of a local channel. TCHD is represented as a rectangular box. The position and possible direction of the lateral migration of the channel is based on extrapolations of the field observation at TCHD. The variations in sedimentary deposits, their thickening and thinning were used to extrapolate possible changes beyond TCHD. (A) A local depression along an abandoned channel that pooled seasonally at a distal part of a floodplain. The environment was stable for long enough time that allowed the establishment of the multi-general forests. (B) A crevasse splay changed the environment from a seasonal pool to a permanent wetland. The forest didn’t survive the change in the depositional environment. (C) The accretion of the clay and silt changed the local landscape which resulted in lateral migration of the local channel. (D) The channel scoured the deposits in the local depression and carved a new channel. (E) Shale deposited on the distal part of the active channel (F) Another lateral change made the bank of the active channel stable as was evident from the development of a paleosol.

Hand samples were saw-cut from the TCHD fossilized forest floor and thin sections were made from the hand samples to investigate the macroscopic and micromorphological paleosol features (Fig 7). Three cross-section profiles of the sediment deposits from the southeast, north, and northwestern walls of the TCHD quarry were made (Fig 8). The purpose of the cross-section profiles was to correlate the quarry walls and reconstruct the changes in the local depositional environments over time (Figs 9 and 10).

## Results

### TCHD quarry floor

#### Section A-B

**Fig 11:**
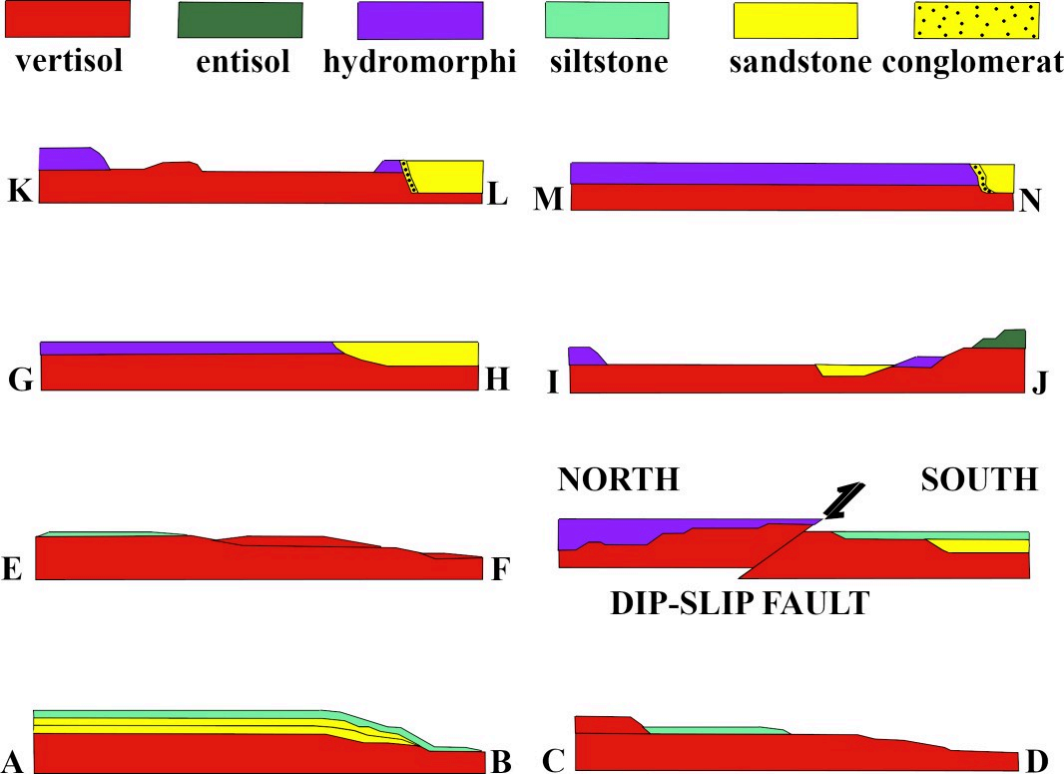
Sedimentary profiles of the cross-sections A-B to M-N. The sedimentary profiles of the cross-sections A-B to M-N were drawn based on the field observations and drill cores. The profiles are not scaled and are meant to complement the map in Fig 2. The variations in sediment sizes, the thickness of the deposits, and planar exposures are shown from sections A-B to M-N.

**Fig 12:**
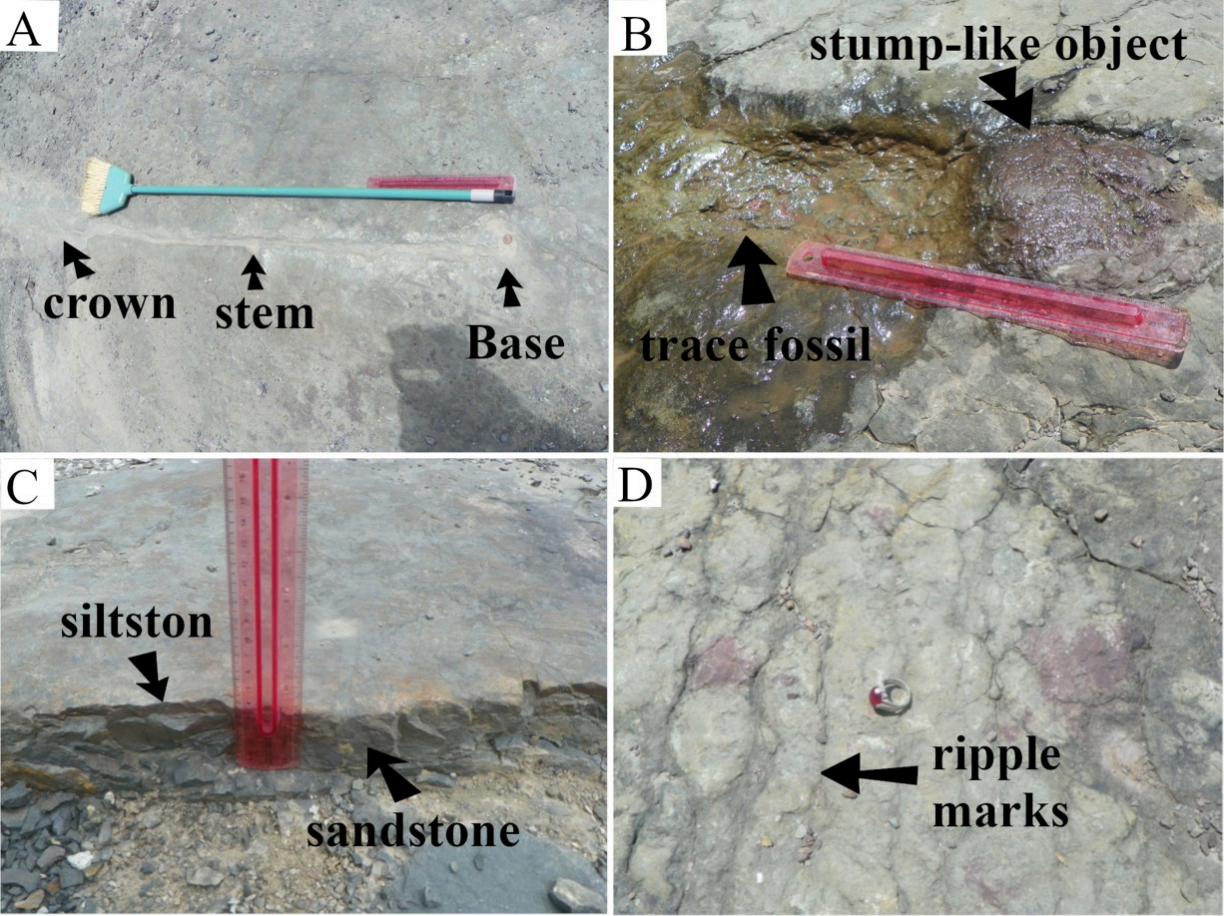
Main features of the A-B section. (A) A washed-in young *Eospermatopteris* tree. (B) The displaced stump-like object in the form of an oxidized cast and its trace-mark in the form of a mold. (C) Organic-rich, fossiliferous sandstone underlying the siltstone. (D) Small scale ripple marks. Note that the direction of the displacement of the object is southward, and the *Eospermatopteris* tree northward. Additionally, the redox features of the siltstone are also notable.

Cross-section A-B was located at the southernmost part of the TCHD quarry floor and oriented in the East-West direction (Fig 2). Close to point B, there was a preserved-terrace that ran from South to around the middle of the quarry. From the middle of the quarry northward, the terraces were scoured and covered by sandstone. The quarry floor at the cross-section A-B consisted of three distinct stacks of paleosols (Fig 11). The bottom-most layer was covered. The second layer was composed of the organic-rich medium-dark-gray (N4) fossiliferous sandstone that was vertically exposed at the preserved terrace. While the thickness of the sandstone varied along the terrace, at its thickest point, it measured five and a half centimeters (Fig 12C). Branching and tapering carbonaceous roots were frequent in the sandstone. The roots were mostly less than a centimeter in diameter. The topmost layer was composed of a very pale olive-green (10YR 8/1) siltstone. The siltstone colors varied. Towards point-A, there was a subrounded stump-like object and a trace-fossil in the form of a round depression right behind it (Fig 12B). Both objects had identical shapes and sizes and their diameters measured around 15 cm. The stump had a medium-dark red color (10R 3/4) and its trace-fossil had a light olive-green color (2.5Y 8/1). The object displaced towards the south. Some scattered medium-dark red patches had scattered distribution in the vicinity of the stump-like object. Around the middle of the cross-section, a trace-fossil of a full-sized young tree measuring around 1.5 meters tall (Fig 12A) made its presence noticeable. The tree had a bulbous base and a crown identical to the *Eospermatopteris* trees. It was oriented in the south-north direction where the base of the tree pointed towards the south and the crown towards the north of the quarry. Small scale ripple marks carpeted the surface of the siltstone (Fig 12D).

#### Interpretation

The organic-rich small planar and trough cross-bedded sandstone was indicative of a crevasse splay deposit at the distal part of a floodplain (Figs 9B and 9C and 10B and 10C). The presence of the carbonized roots and the absence of redox features suggested that the local depression remained inundated for a considerable amount of time. Based on the presence of both plant roots and the primary sedimentary structures, the sandstone could be categorized as a hydromorphic protosol. The long duration of inundation was also supported by the fact that the sandy-soils drained relatively faster and couldn’t remain saturated for long. The olive-green siltstone and the ripple marks indicated a change in the drainage pattern. The olive-green color of the paleosols matrix was associated with the reduction of metal ions such as iron. The red colors of the stump-like object and some small patches pointed to the oxidation. Along with ripple marks, the redox features showed that the depth of flood water decreased over time. The trace fossil of the full-sized young tree suggested the settlement of water-carried debris and its orientation pointed to the direction of the water movement, also known as paleocurrent direction. Interestingly, the fallen tree pointed towards the north and the stump-like object displaced southward. The opposite directions of the paleocurrents suggested two stages of flooding, the initial northward flooding that deposited washed-in organisms and the southward direction of the receding water. The direction of the small-scale ripple marks and surface paleosol redoximorphic features provided additional support for this interpretation.

#### Section C-D

**Fig 13:**
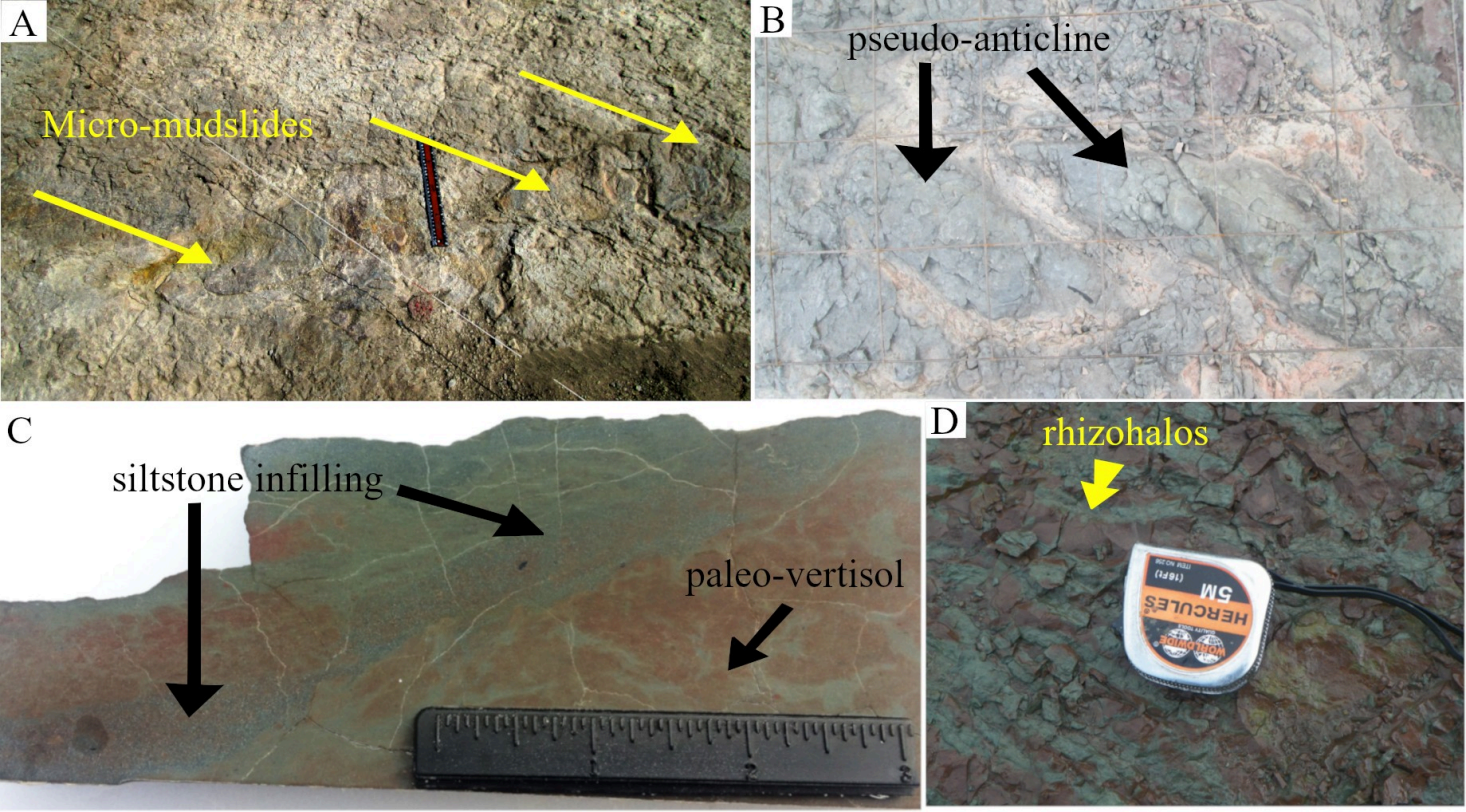
Some informative features of the paleo-vertisol along the C-D section. (A) Multiple-steps mudflow at the bank of the preserved abandoned channel. The preserved mudflow was located between Arch-5 and Arch-6. Note yellowish-brown oxidation coloration at the lower end of mud-flow. (B) Pseudoanticline: Note the visible micro-lows around the pseudo-anticlines. Black arrows point to the micro-highs. (C) A polished section from a cut-saw hand-sample from surface paleosol: Fine sand and silt size sediments filled a desiccation crack (fine sand and silt size sediment capped the forest floor and preserved it). (D) Light-olive green rhizohalos-like features in the weak-red paleosol matrix on the exposure of the capping paleosol close to the point-C: Note they have some of the features of the *Eospermatopteris* rhizohalos such as being equidimensional, non-branching and running in bundles.

**Fig 14:**
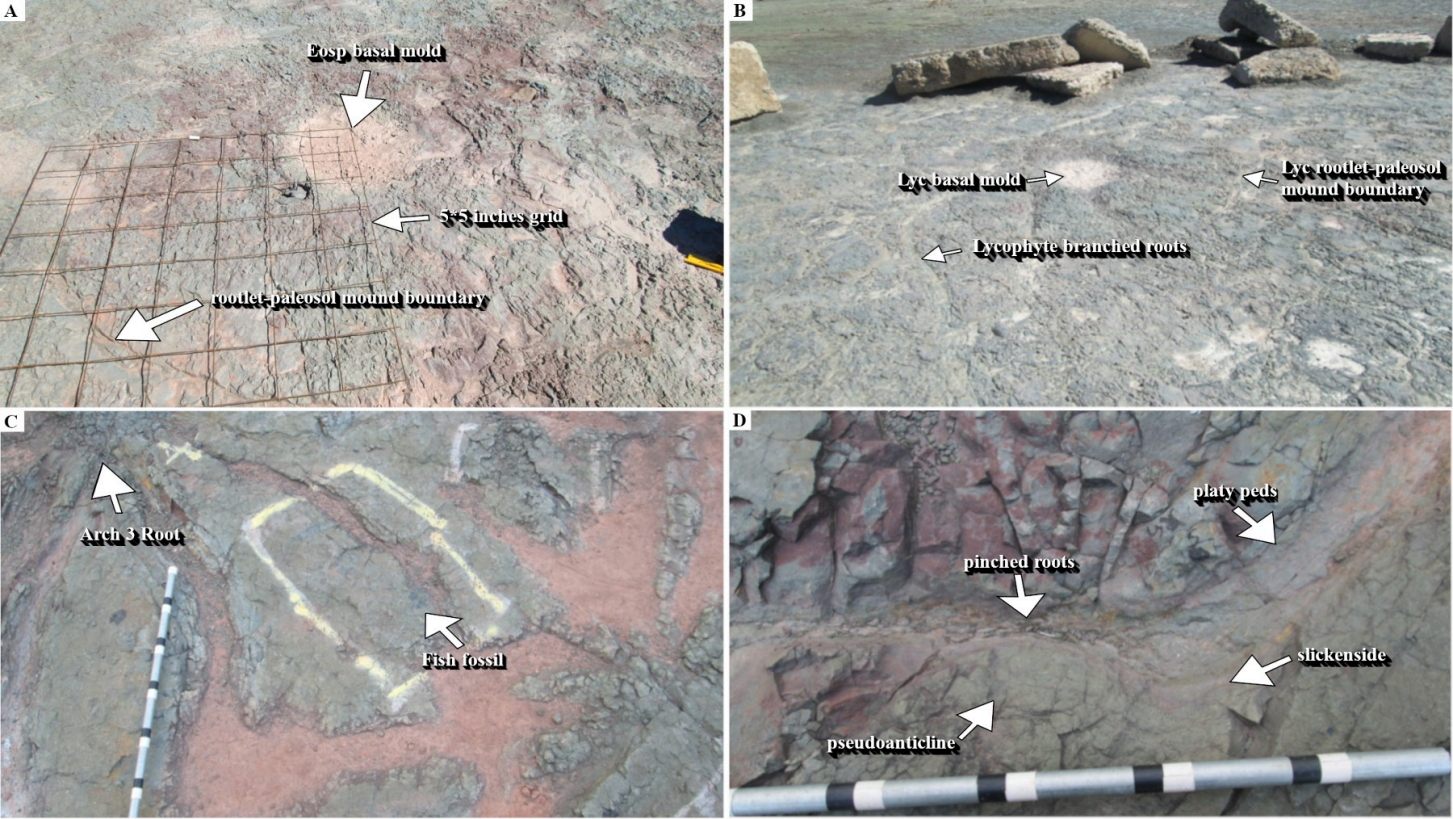
Representative fossils of roots systems belonging to three Middle Devonian tree clades. (A) *Eospermatopteris* stump-molds composed of a central depression surrounded by a raised mound of paleosol-rootlets. The mound is covered with rootlet rhizohalos. Note the slickensides at the boundary between the paleosol-rootlet mound, and the paleosol-matrix. The grid used to map the rooting system is also visible in the picture. (B) The lycopsid root system was composed of *Eospermatopteris-*like stump mold and *Archaeopteris*-like branched roots. (C) Proximal roots of Arch-3 and a trapped fossil of fish that was marked with yellow color for the extraction. (D) A pinched root of *Archaeopteris* between pseudoanticlines. The slickensides and platy peds that developed around the root are notable features of root-paleosol interactions.

The section C-D passed through an area of the TCHD where the preserved forest floor had the best exposure and had been the focus of most of the studies at this site (Fig 3). Based on the tree root fossils, it was estimated that the site had a tree density of 20 in approximately 2000 m^2^ area. As parts of the forest floor were still covered, the density of the trees might be higher. A section of the capping paleosol -sediments that covered the forest floor and preserved it- with strong redox features was the most prominent feature at the point-C (see S3 Fig via https://doi.org/10.6084/m9.figshare.14908014.v1). Redoximorphic platy peds dominated the lower section of the exposure which transitioned into medium-sized, angular blocky peds. The peds had mostly weak-red (10R 5/3), and their boundaries light cyan-green (3G 7/5) colorations. What appeared to be bundles of light cyan-green rhizohalos were also observed on the mound surface (Fig 13D, see S3 Fig via https://doi.org/10.6084/m9.figshare.14908014.v1). The bundles were equidimensional and non-branching which were main features of the *Eospermatopteris* rootlets. However, they weren’t attached to a stump or trace fossil of a stump. The rhizohalo-like objects and redoximorphic features were reoriented in a semi-circular manner by slickenside on the surface of the exposure (see S3C Fig via https://doi.org/10.6084/m9.figshare.14908014.v1). Slickensides were also observable in vertical exposures of the capping paleosols (see S3B & S3C Fig via https://doi.org/10.6084/m9.figshare.14908014.v1). Close to the point-C, there was also an exposure of the capping paleosol at the base of the southeast wall (see S3B Fig via https://doi.org/10.6084/m9.figshare.14908014.v1). The lower part of the exposure was dominated by the redoximorphic platy peds which transitioned into olive-green peds in the upper half of the paleosol. No fossils of tree roots were observed in the vertical exposures of the capping paleosols. Walking down the exposure of the capping paleosol, a thin layer of very pale olive-green siltstone had partially covered the paleosol surface and hosted numerous fish fossils (7C). Close to the point-C, there were also several trace-fossils of the *Eospermatopteris* tree stump-molds spread almost along a line from the south to the north of the C-D section (Fig 3). Subhorizontal rhizohalos and rhizoliths covered the rootlet-paleosol mounds of the *Eospermatopteris* fossils that extended beyond the rootlet-paleosol mound into surrounding paleosols (Fig 4). With exception of the Eosp 1, Eosp 2–4 were located south of the C-D section (Fig 3). Eosp 1’s central depression and rhizolith in the rootlet-paleosol mound had light olive-green (2.5Y 8/1) coloration. The Eosp 2–3 had identical redoximorphic features, where their central depressions had light olive-green color and the surrounding rootlets-paleosol mounds were composed of the weak-red sub-angular peds. The shapes of rootlets-paleosol mounds didn’t follow the geometry of the central depressions and showed directionality in the form of the bulging towards the southeast of the quarry (Figs 4 and 14A). Additionally, the developments of the multiple slickensides at the boundary between the rootlets-paleosol mounds were noticeable. Like rootlets-paleosols mound, the slickensides showed directionality. Rootlets had been preserved mostly in the form of light olive-green rhizohalos, and also some rhizolith casts. The rhizohalos were almost equidimensional, non-tapering, and had 360-degree distributions (Fig 4). While *Eospermatopteris* stump-molds varied in their sizes, the central-depression of the large fossils measured around 50 cm in diameter. Depending on the degree of bulging, on average, the rootlet-paleosol mound extended from central-depression to the paleosol-boundary around 30–50 cm.

Several well-preserved fossils of lateral roots were exposed along the C-D section (Fig 3). These root systems shared the common morphology of modern trees such as branching and tapering. Arch 1–2 and likewise, Arch 3–4 were coupled-root systems, meaning their terminal roots overlapped. Arch 5 and 6 were solitary root systems. These root systems are the first such well-preserved fossils of *Archaeopteris* trees that so far have been discovered. The lateral roots branched out into multiple suborders from proximal to distal parts of the root systems. With 11 main roots, Arch 5 was the most well-preserved root system at the TCHD, and was located at the proximity of the point-D. Some of the main roots of Arch 5 branched into 11 suborder roots. Most of the root systems were preserved as mold fossils (Fig 14C and 14D). Parts of the terminal roots were preserved as casts and carbonaceous rhizoliths. The branching behaviors were localized meaning, there was no apparent common branching rate among the main roots. For instance, a 4 meters long main-roots branched out into 6 sub-orders, while a meter-long main-root into 11 sub-orders. Some of the main roots measured up to 7 meters long. In addition to the branching and tapering, the directional distributions of the branches were the most obvious features of the *Archaeopteris* root systems at TCHD. For example, if we divided the root systems of the Arch-5 into the four geographical coordinates, 6 out of the 11 main-roots of the Arch-5 would fall in the southwest coordinate. It appeared that the main-roots avoided the microhighs of the pseudoanticlines by following the trends of microlows (Fig 13B). The pinching of one of the main roots by the pseudoanticlines illustrated the challenges that roots faced by the hardening of the paleosol surface features (Fig 14D). Peds coloration noticeably changed from Arch-1 to 6, respectively. The surface paleosol around Arch-1 and 2 had mixed weak-red and light olive-green coloration. Light olive-green dominated around Arch-3 and 4 areas. Most of the fish fossils were recovered from the olive-green peds and overlying thin-layer of siltstone. The apparent trapping of the fishes in-between roots of Arch 3 and 4 was an interesting event preserved at the TCHD. The areas covered by the olive-green siltstone, the mean features of the surface paleosols such as desiccation cracks and pseudoanticlines were noticeably not visible. The surface color of paleosol around Arch-5 varied from medium-dark-gray (N4), gray (5Y 6/1), yellowish-brown (10Y 5/8), to brownish-yellow (10Y 6/8). Arch-6 occurred on a preserved-terrace where the surface-peds colors ranged from yellowish-brown to brownish-yellow. The most notable difference between the fossils of the *Eospermatopteris*-rootlets and *Archaeopteris* root networks in the C-D section area was their mode of preservation. *Eospermatopteris* rootlets were dominantly preserved in the form of the rhizohalos and in contrast, the roots of the *Archaeopteris* were preserved dominantly in the form of molds but also in the form of the casts and some carbonaceous rootlets. Macro-scale slickensides were observed along with the main roots of Arch-3 and 4. The peds along the main roots and also the terminal roots were platy and longitudinal. Other than the immediate vicinity of the roots, most of the peds along the C-D section were angular and subangular medium sized blocky peds.

Parallel to the Arch-5, a distinct fossilized root system with unique morphology identified as a possible lycopsid tree [11]. The lycopsid fossil showed characteristics of both *Eospermatopteris* basal-molds and the branching networks of the *Archaeopteris* root system (Figs 3 and 14B). The central-depression, rootlets-paleosol mound were the common morphology it shared with *Eospermatopteris* stump molds. Multiple main roots directly connected to the central-depression cut through the rootlets-paleosol mound and showed strong directionality towards the southwest. The main root branched and tapered but the tapering and branching rates were very low compared to the *Archaeopteris* roots. The main roots branched into a couple of suborders towards the distal part of the root system. Just like Arch-5, most of the root-system occurred in the medium-dark-gray area and the distal parts of the root-system extended into the preserved-terrace that had dominantly yellowish-brown to brownish-yellow surface-peds. The central-depression of the lycopsid base-mold fossil measured around 56 cm (59 cm at the widest part) and one of its roots measured around 8 meters long.

All the mapped pseudoanticlines occurred at the medium-dark-gray areas. As previously noted, the areas covered with olive-green siltstone had very poor exposures of the pseudoanticlines. Pseudoanticlines had sub-oval microhighs- one side was broader than the opposite side (Fig 13B). On average, the broader side of the pseudoanticlines pointed towards the southwest of the quarry. The pseudoanticlines varied in size and showed multi-generational developments. Several smaller second-generation pseudoanticlines had developed inside the regular pseudoanticlines. A regular pseudoanticline had well-developed microlows that separated it from the neighboring pseudoanticlines. Light-olive-green platy and longitudinal-peds are oriented along the microlows of the pseudoanticlines. The size of the pseudoanticlines in the non-rooted area decreased from PA-1 to PA-2 and increased again in PA-3 (Fig 5). In rooted areas, the pseudo-anticlines of PA-4 that were located in the proximity of the lycopsid fossil were relatively smaller and numerous than pseudoanticlines of PA-5 that were located in the proximal area of Arch-5. Likewise, the pseudoanticlines of the PA-5 were relatively larger than PA-6 which were associated with the distal parts of the Arch-5. Close to the terminal roots of the Arch-5, there was a meter long preserved multistep mudflow (Fig 13A). The width of mudflow varied.

Hand samples were cut-sawed from surface paleosol in the proximity of the root systems that varied in surface-ped coloration from weak-red, olive-green to medium-dark-gray. The cut-samples helped in observing the vertical changes at the surface paleosol. The common differences among the samples were the depth at which redoximorphic features appeared and the mode of rootlet preservations, in the form of the rhizohalos and rootlet-casts. In local areas around Arch-1 and 2, the redoximorphic features appeared at just a few centimeters depth. Very fine medium-dark-gray sandstone and olive-green siltstone filled the surface cracks (Fig 13C). In the hand samples collected from the proximity of Arch-3 and 4, both rhizohalos and rootlet-casts were olive-green while matrix paleosol had pale-red (10R 7/3 and 10R 7/4) coloration. The paleosol in close vicinity of the rhizohalos and rootlet-casts had the lighter hues of red (10R 6/8) and became paler further away from the rhizohalos. In addition to the light coloration, the macro-scale peds—millimeters in diameter- also dominated the areas around the rootlets. The macro-scale peds were mostly semi-spherical. Some of the hand samples showed sub-angular and tapering pale-olive green root-casts preserved in pale-red paleosol matrix. Additionally, medium-dark-gray rhizohalos in the yellowish-brown matrix were also observed in the hand samples. Thin-sections made from the hand-specimen showed illuviation of clay of the crack fillings, voids, and structures that appeared like burrows. The burrow-like structures were mostly equidimensional and non-branching. Likewise, abundant iron-oxides globules and concretions were observed. The iron-oxide concretions formed around micro-peds (Fig 7C) and large sediment particles. While clay-sized sediments were dominant, organic materials and clay-sized sediments increased from Arch-1 to Arch-5. Spores and fine roots were other common features of the micromorphology of the paleosol (Fig 7A, 7B and 7D). While the sediment particles in the olive-green siltstone were well-rounded, the differences in the particle sizes between siltstone and underlying paleosols were contrastingly different.

A 1.6 meters drill-core extract along the C-D cross-section revealed two stacks of the paleosols with identical features (Fig 6A–6F). The dominant features of the paleosols included a reduced or bleached zone (Fig 6A) followed by a zone of rhizoliths and mottling (Fig 6B) where the color of the paleosol matrix was dominantly pale-red. The color of the paleosol-matrix transitioned into light red with increasing depth, and likewise around the bundles of the rhizoliths. The rhizoliths were composed of light-red or pale-red cores surrounded by the light-olive green rhizohalos. The rhizoliths ranged from very fine (less than 10 mm in diameter) to fine (10 to 20 mm in diameter). Some of the rhizoliths branched downward (Fig 6F). Some drill-core extracts (NYSM-13) showed no desiccation-features. But there were core extracts (NYSM-4, for instance) that showed a few centimeters thick zones of the shrink-swell where the pale-red peds of different sizes—ranging from very fine (less than an mm) to very coarse (more than 10 mm)- suspended in a light-olive-green matrix (Fig 6H). The peds were mostly subrounded. The olive-green matrix also hosted some small pieces of organic materials. Another important difference between the core-extracts of points C and D were the percentages of the mottling and rhizoliths where the percentage of the mottlings were less than 50% in the former and more than 50% in the latter.

#### Interpretation

The area around the C-D section revealed a wealth of information about the composition of the extinct forest, the paleosol that made the forest floor, the depositional environment, local topography, drainage patterns, and the preservation of the fossilized landscape. The most important finding was the discovery of branched root systems that branched several orders from the proximal to the distal parts of BN. The only trees known from the Devonian period that had complex branched root systems were *Archaeopteris* trees. The roots of the lycopsid trees also branched but were limited to dichotomous branching. The possible lycopsid tree root found at the TCHD had an interesting intermediary root system that shared the main features of both *Eospermatopteris* and *Archaeopteris* tree root systems. The dominant features of the paleosols such as blocky peds, slickensides, desiccation cracks, and pseudoanticlines were all associated with swell-shrink as a result of seasonal wetting and drying processes. The swell-shrink features are the chief characteristics of the vertic soils that are rich in expansive clay minerals such as smectite. By repeated cycles of wetting and drying smectite minerals change into illite. Based on the vertic features and high concentration of the illite, the paleosol was identified as paleo-vertisol. The repeated wetting-drying cycles resulted in hardening of the peds which in turn led to the development of the permanent surficial features such as multigenerational pseudoanticlines. The pseudoanticlines posed challenges to the root systems. *Archaeopteris* roots avoided the drier microhighs and followed the wetter microlows. Thicker roots got pinched in the narrow microlows as they grew which suggested that microhighs were permanent hardened surficial features. Redoximorphic features of the paleosols were associated with the drainage patterns. The redoximorphic features were dominant in the surface-paleosol in well-drained parts of the paleosol. In comparison, in poorly drained areas, the redoximorphic features appeared at subsurface paleosols which indicated pooling of water at the soil surface that lasted relatively longer in the poorly drained areas. The dominant drainage pattern also strongly influenced the root behaviors of the *Archaeopteris* trees and likewise the size and geometry of the pseudoanticlines. The strong directional growth of the *Archaeopteris* roots towards the southwest, the orientation of the pseudoanticlines with their broader sides pointing towards north and northeast of the TCHD, and also the change of the redoximorphic features from dominance of iron-oxide to pyrite oxide from point-C to the point-D suggested the presence of a local slope along the C-D section. Furthermore, the preserved terrace, multiple-step micro-mudflow as well as the oxidation of pyrites that gave paleosol the yellowish-brown coloration at point D collectively suggested the presence of a local abandoned channel that remained saturated longer than the point C. The root fossils at point C were mostly in the form of the fossil-molds. At point-D, there were root-casts and carbonaceous rootlets which implied relatively deeper burial. This difference in the modes of preservation also agreed with the presence of the local slope.

The *Eospermatopteris* fossils had two morphologically distinct parts, a central depression, and a rootlets-paleosol mound. The central depressions were clear footprints of the bulbous bases of the trees and the mound very likely formed by pushing out the paleosol during the expansion of the bulbous bases as the trees grew. The rootlets-paleosol mounds worked as a single unit to anchor the trees to the ground. The changes in surface paleosols from light-olive green to red weak and olive-green between Eosp-1 to the Eosp-4 suggested the presence of a rising slope from south to the north of the C-D section. The absence or burial of pseudoanticlines at the boundary of the Eosp-4 and the host paleosol, and in contrast, the presence of the slickensides at the boundaries of the Eosp 2 & 3 also agreed with the presence of a rising slope at this area. Furthermore, the thinning of light-olive green capping siltstone from south to the north of the C-D cross-section, as well as the entrapment of most of the fish fossils along C-D cross-sections, were all provided independent lines of evidence for the presence of a rising slope.

The chroma of the paleosol along the C-D section decreased from east to west, increased from south to the north, and also with the depth of the soil. The changes in chroma indicated that the local mounds or ‘local highlands’ along the C-D section didn’t remain saturated for more than a few days but local channels remained saturated for several months each year. Furthermore, the paleosol saturation was not the result of the shallow water table but the pooling of surface water runoff at the local channel. In general, the abandoned local channel worked as a seasonal wetland such as modern vernal pools. The clay size particles worked as a barrier to seepage or percolation of the water to the depth. This barrier also kept paleosol saturated from a few days to months depending on the location of the paleosol in the landscape. The large sizes of the root systems suggested a long duration of stable environment at TCHD. The two stacks of well-developed paleo-vertisols revealed by the 1.6 meters long drill core extracts provided further evidence of a long stable landscape at the TCHD. The presence of rhizohalos all along paleosols showed that the water table was quite deep, and other than the seasonal pooling of water at the surface paleosol, it was mostly dry land. Based on the long duration of the stable land, clay-sized particles, and seasonal pooling of the local channel, it could be inferred that the depositional environment was a distal floodplain. The presence of the contrasting rooting systems of the two extinct trees of the Devonian Period along a lycopsid tree on a seasonal wetland environment made it obvious that the earliest forests of the Middle Devonian New York State were very adaptive in the semi-arid climate and the seasonal wetlands were very important refugia for all the trees. The presence of the branched rhizolith in the stacks of the two paleosols showed the multigenerational forest at the TCHD.

The capping sediments at the C-D section provided additional information about the crevasse splay events that preserved the forest floor. Unlike the A-B section where the organic-rich sandstone transitioned into siltstone with ripple marks made the bulk of the capping sediment, at the C-D section, it was a thin layer of olive-green silestone that capped the forest floor. This was an indication of a rising slope from sections A-B to C-D. Furthermore, the washed-in fossil at the A-B section was a young tree and fossils at the C-D section were numerous disarticulated fish fossils trapped in the proximal roots of the Archaeopteris trees. The fossils complemented the evidence for a rising slope. The younger tree that was heavier got deposited in the A-B section and lighter disarticulated fishes were carried away further and got trapped by the larger proximal roots of the trees. The thin layer of the capping sediments and fossils supported a flooding event at the TCHD. The capping paleosols provided information on the events that followed the flooding event. The lower portion of the capping paleosol was dominated by the redoximorphic platy peds which indicated the vertical accretion of the fine-grained sediments. The vertical accretion of the sediments suggested a change from non-depositional to depositional environment such as proximal floodplain. The presence of slickensides and reorientations of the redoximorphic features suggested the seasonal wetting and drying by rise and fall of water level at the local highland at the C-D section. The presence of what appeared to be a bundle of enigmatic rhizohalos on the top of the lower section of the capping sediment (see S3C Fig via https://doi.org/10.6084/m9.figshare.14908014.v1) was an interesting phenomenon. The rhizohalos possessed most of the features of the rhizohalos such as being equidimensional, non-branching, exploring the sediments by snaking in different directions both vertically and horizontally in the form of a bundle. Since, we couldn’t associate it with any stump and similarly, no rooting were observed in the vertical exposures of the capping paleosol (see S3A & S3B Fig via https://doi.org/10.6084/m9.figshare.14908014.v1), we couldn’t be certain that these features were actual rhizohalos. Furthermore, it was possible that the reorientation of the redoximorphic features by the slickenside (see S3C Fig via https://doi.org/10.6084/m9.figshare.14908014.v1) created these features. The semi-circular reorientation of the redoximorphic features at horizontal, and the bulging at the vertical exposure could be best explained by the analogy of a horizontal section of an onion bulb. From top-view, the scale leaves of an onion bulb appear as concentric rings. In similar fashion, the concentric rings of the redoximorphic features (see S3C Fig via https://doi.org/10.6084/m9.figshare.14908014.v1) from the top-view, and the bulging from the side-view (see S3A & S3B Fig via https://doi.org/10.6084/m9.figshare.14908014.v1) could be explained as a 3D view of a slickenside. Based on the platy peds, redoximorphic features, slickenside and absence of the extensive rooting, the capping paleosol at the C-D section was categorized as moderately developed paleo-vertisol. The transition of the redoximorphic platy peds to the olive-green wedge peds indicated a change in the landscape. The accretions of the sediments over time filled the local depressions which created a more uniform landscape which resulted in poor-drainage of the landscape.

#### Section E-F

**Fig 15:**
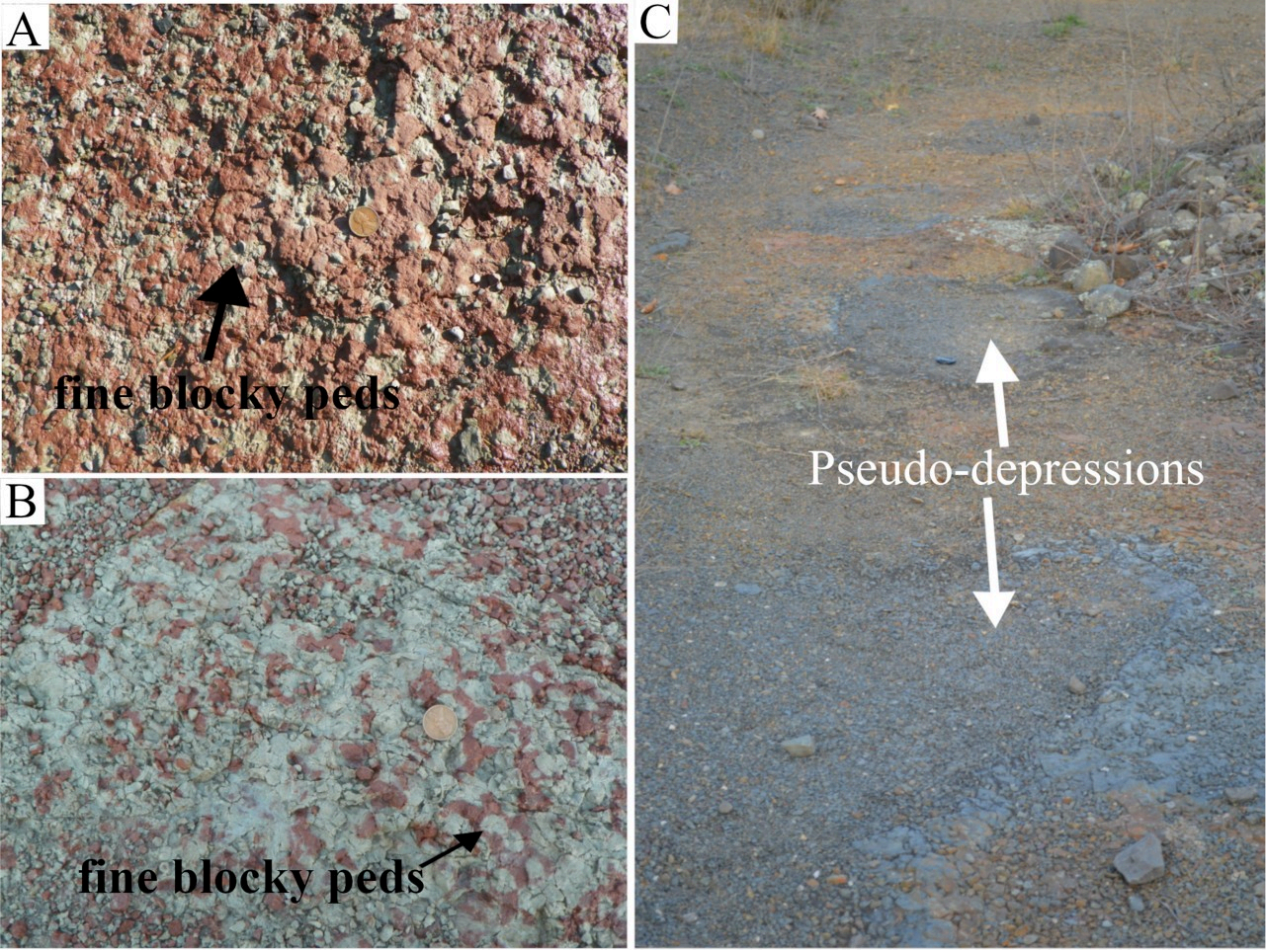
Some main features of paleosol along the E-F section, and surrounding areas. Figures (A) and (B) show granular peds of similar sizes. Note that in figure (A) weak-red peds make the matrix and in figure (B), the light-olive green peds make the matrix of the paleosol. (C) The area between section E-F and G-H shows a linear distribution of the pseudo-depressions that mark the edge of the local-depression.

The point-E and the surrounding areas were mostly covered. The point-F and the surrounding areas were well exposed and mostly composed of the well-developed fine-size (5 to < 10 mm diameter) blocky-peds (Fig 15A and 15B). The weathered peds became granulated. The color of peds varied in the exposed areas from the middle of the E-F section towards the point-F. Weak-red was the dominant color among the peds. The light-olive green peds dotted the weak-red paleosol matrix around the middle part of the E-F section. More towards the point-F, the pale olive-green peds made the bulk of the surface paleosols, and the weak-red peds dotted the paleosol matrix. Closer to point-F, the yellowish-brown peds became dominant.

#### Interpretation

The well-developed fine size blocky-peds and the change in the coloration suggested the intensive swell-shrink and root activity along a local slope. The blocky peds formed as a result of the swell-shrink processes in a paleosol with high clay content. It was very likely that root activities broke down the peds. The well-developed peds facilitated the water drainage and also posed less resistance to the roots. The well-developed ped and the change in the coloration of the peds from weak-red to pale-olive green and yellowish-brown at a short distance suggested the presence of a second local paleosol mound.

#### Fault-line

Almost around the middle of the TCHD, there was a dip-slip fault-line (Figs 2 and 11) where the northern half of the quarry floor had been uplifted approximately 30 cm. The fault-line exposed a vertical section of the paleo-vertisol as well as a thin layer of the capping sediment. The thickness of the capping sediment was a few centimeters at the faultline. Its light-gray (5YR 8/1) color made it distinct from the underlying paleosol. The vertical section along the faultline revealed medium size (20 to 50 mm in width), light olive-green color prismatic peds that showed no evidence of rhizoliths. The prismatic peds transitioned into fine size (5 to 10 mm) blocky peds. The weak-red blocky peds made the bulk of the underlying paleosol while the light-olive green peds dotted the paleosols.

#### Interpretation

The climate-induced unloading of the thick ice sheets towards the last glaciation of North America resulted in the rebounding of the lithosphere at this part of the world and hence in the developments of the local faults. The dip-slip fault at the fossil site was one of such local faults. The fault revealed the highest point of the local rise. Prismatic peds are common where swelling and shrinking [25] are extensive such as local highlands. The presence of the prismatic peds followed by blocky peds suggested an increased concentration of the clay on the local rise and absence of the root fossils suggested that this part of TCHD was barren. The reappearance and change of the capping sediment from light-olive green to the light-gray indicated the beginning of a local depression that dipped northward.

#### Section G-H

**Fig 16:**
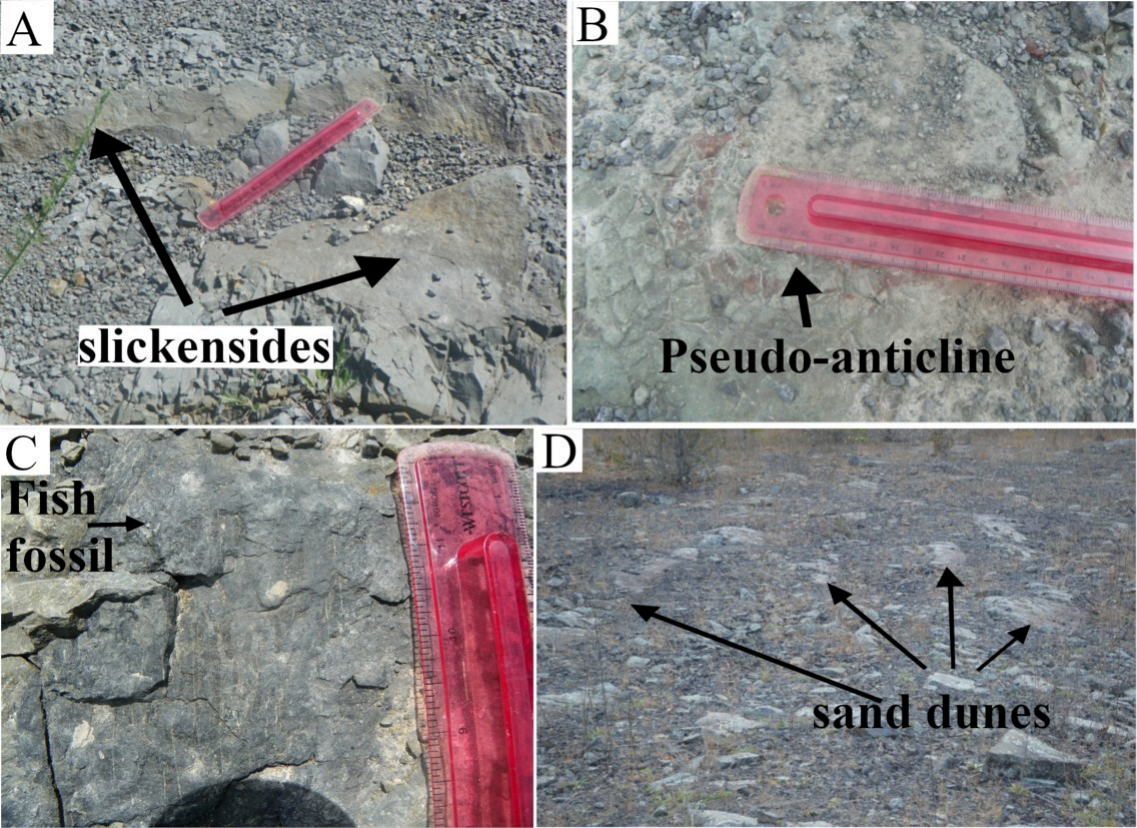
The main features of the paleosol along the G-H section. Interestingly, the areas along G-H sections have both vertic and hydromorphic features. (A) A slickenside at the paleosol surface (vertic feature, also young paleosol). (B) A pseudo-anticline (vertic feature) with oxidized boundary (hydromorphic features). (C) A fish fossil that helps correlate it with light-olive green siltstone at the C-D section. (D) The sand dunes deposit overlying the hydromorphic paleosol.

The G-H section was composed of two different deposits. From point- H to the middle of the section, a light-gray paleosol that bore iron-manganese nodules as well as fish fossils covered the area (Fig 16C). Surface slickensides and pseudoanticlines were other important features of the paleosol. The area between G-H and E-F sections showed large surface slickensides (Fig 16A) as well as some pseudoanticlines (Fig 16B). The microhighs of the pseudoanticlines had olive-green, and their microlows weak-red colors. A small area where the surface-paleosol had been quarried exposed the underlying redoximorphic paleosol. From the middle of the section to point-H, two-dimensional sand-dunes had replaced the paleosols (Fig 16D). The sandstone deposits had erosional boundaries with the paleosol and did not show any ripple marks on them. The stoss-sides of the dunes were orientated northwest and their lee-side southeast. Several pseudo-depressions queued almost in a straight line from the east to the west (Fig 15C) between the G-H section and the fault-line. The shapes of the depressions ranged from spherical to ellipsoid and their small diameter ranged from 15 to more than 30 cm. The boundaries of these pseudo-depressions and the host paleosol showed slickenside developments. A ring of platy ped formed several centimeters thick on the edges of the pseudo-depressions. The platy peds orientations followed the shapes of the pseudo-depressions. The peds close to the boundary were mostly sub-vertical and peds towards the center of the depression mostly subhorizontal. Blocky peds filled the center of the pseudo-depression. The depressions that had lost their platy-peds to erosion showed smooth surfaces that were darker in hue than the host paleosol.

#### Interpretation

The iron-manganese nodules and the absence of the redoximorphic features suggested that paleosol remained saturated for a very long time. The large surface slickensides, as well as the absence of the plant fossils, implied a relatively young age of the paleosol, meaning the paleosol had not enough time to develop permanent features that were characteristics of mature paleosols. The pseudoanticlines with oxidized microlows indicated brief drying up of the wetland. The fish fossils and also underlying paleosol with blocky-peds and redoximorphic features helped in the correlation of this paleosol with the capping sediments such as olive-green siltstone and underlying organic-rich sandstone along A-B and C-D sections that capped the paleo-forest-floor in the south of the TCHD. In the light of changes in the grain size and the paleosols features of the capping sediment from A-B to G-H sections, it became clear that the A-B and G-H sections were part of two separate local depressions which were separated by a local rise that stretched from C-D to E-F sections. The direction of sediment transport was from the south to the north of the quarry. The pseudo-depressions formed as a result of the swell-shrink process at the southern boundary of a local depression where the swell-shrink process happened frequently. At the southern edge of the local depression, the paleosol was thinner and more sensitive to the fluctuations of the moisture contents of the paleosol. Based on the similarity of the shapes and also the occurrence of the platy-peds at the boundaries of the pseudo-anticlines, it became evident that the pseudo-depressions were initially formed as pseudoanticlines. As the swell-shrink was frequent in this area, the peds in the pseudo-anticline broke down and washed away over time leaving behind the trace-fossils of the pseudoanticlines or pseudo-depressions. Fresh weathering of pseudoanticlines also have created recent pseudo-depressions (see S2A & S2C Fig via https://doi.org/10.6084/m9.figshare.14558454.v2). The sandstone dunes suggested a change in the depositional environment from the proximal floodplain to the active channel (Figs 9D and 10D). Because of the local depression, it was very likely that the eastern bank of the abandoned channel at this location had poorly developed terraces or no terraces hence the sand-dunes that deposited by the lateral shifts of channel incised parts of hydromorphic paleosol and likewise underlying paleo-vertisol. The boundary between sand-dunes and paleosols, as well as the orientation of their stoss-side, made it possible to infer the direction of the paleocurrent. The active channel that scoured the paleosols flowed from north to south.

#### Section I-J

**Fig 17:**
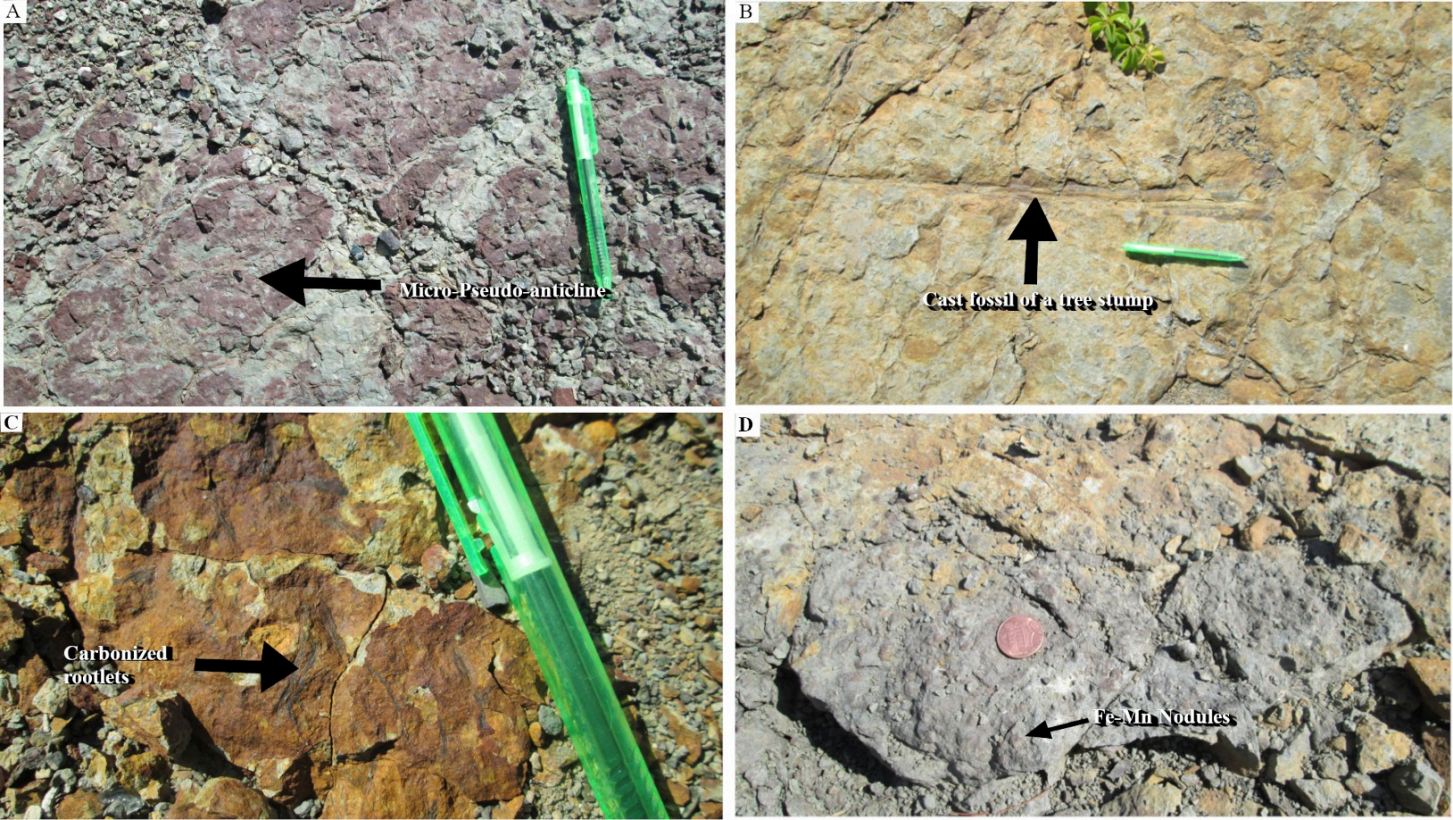
Main features of the paleosol underlying hydromorphic paleosol. The paleosol underlying hydromorphic-paleosol was exposed by the quarrying at the local-depression. The presence of the fossilized roots, stems, blocky peds, and pseudo-anticlines as well as its stratigraphic position (underlying light-gray hydromorphic paleosol) made it correlatable with paleoforest at the C-D section. (A) Mini-pseudo-anticlines with oxidized microhigh (weak-red) and reduced microlows (light-olive green): Located between sections I-J and K-L. (B) A cast-fossil of a stem: Note the broad-base and the tapering of the stem. (C) Some carbonized impressions of the branched rootlets. (D) Two hydromorphic features, the yellowish-brown coloration of the surface paleosol (as a result of pyrite oxidation) and iron-manganese in the subsurface paleosol. Note that the paleosol features such as pyrite oxidation, iron-manganese nodules, and carbonized rootlets all indicating that ponding happened at this location, as a result of the impervious subsurface soil as were evident from the presence of the iron-oxide coloration of the micro-pseudoanticline in the subsurface paleosol.

The I-J section showed the highest diversity of paleosols and sediments from the point-I to the point-J. Point-I had a vertical exposure of two stacks of paleosols. The underlying paleosol showed poor horizonation. It had prismatic and blocky peds and likewise showed redoximorphic features. Pale-olive green prismatic-peds made the bulk of the top horizon. Yellowish-brown cutanes coated the surfaces of some prismatic peds. Blocky peds of light-olive green color, and heavy slickensides made the main features of the subsoil. The overlying paleosol also showed horizonation. The top-horizon was composed of light-gray bulky peds (featureless horizon) that bore abundant iron-manganese nodules. The subsoil was composed of light-gray wedged-shaped peds. The thickness of the section, as well as paleosols, varied along the paleosol exposure. In the area between I-J and K-L sections, the quarrying had uncovered the subsoil of the underlying paleosol in this section. The plan exposure of the subsoil was mainly composed of micro-pseudoanticlines (Fig 17A). The quarrying had leveled the microhighs, however, the shapes and the boundaries of the pseudoanticlines were recognizable. The pseudoanticlines were composed of weak-red blocky peds and their boundaries of light-olive green blocky and platy peds.

**Fig 18:**
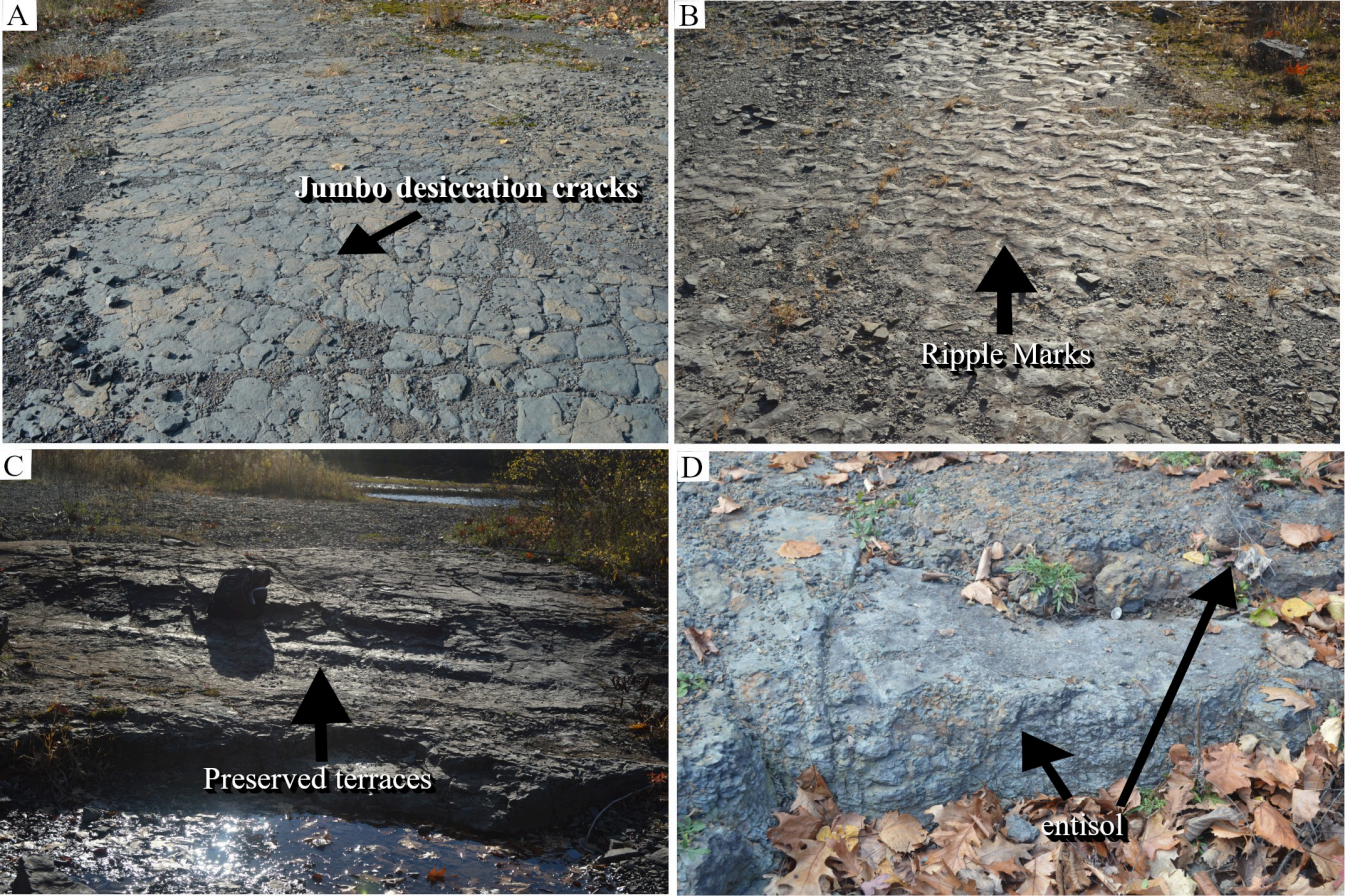
Preserved features of the abandoned-channel along the I-J section. (A) The wedged-shaped jumbo-desiccation cracks are oriented parallel to each other on a locally preserved mound. Regular desiccation cracks of varying shapes and sizes developed inside the jumbo cracks. (B) Sinuous-crested ripple marks on the channel floor. (C) A preserved terrace on the left bank of the local channel. (D) Paleosols with light-olive green peds on the left bank of the local channel. Note the absence of root fossils of any type.

The middle part of the I-J section was covered. The rest of the section was exposed as a result of a creek formed by the displacement of northwestern walls. A left-lateral strike-slip fault (Fig 2) where the northern local block (included parts of northwest, north, and northeastern walls) had moved left past the southern block (included parts of northeastern and northwestern walls). At around 3/4th of the section, a preserved terrace was covered by mud-cracks as well as sandstone casts of the desiccation cracks. The terrace sloped towards the west of the quarry and ended in a preserved channel floor. Light-gray wedged-shaped peds had covered the channel floor at this part. Close to point-J, several preserved landscape features with primary sedimentary structures made their existence noticeable. Jumbo-desiccation cracks carpeted the surface of a preserved local paleosol mound (Fig 18A). The wedged-shaped jumbo-desiccation cracks oriented parallel to each other in the east-west direction along the downslope of the paleosol mound. Small-scale desiccation cracks with shapes ranging from polygonal, square, spherical, and wedged-shaped depending on their location filled the jumbo-cracks. No sign of the redoximorphic coloration was observed at the boundaries of the desiccation cracks. The light gray was the prevalent color of both jumbo-cracks and small-scale desiccation cracks. The preserved paleosol mound transitioned into a flat area that was covered with sinuous-crested ripple marks (Fig 18B). The stoss-side of the ripple marks faced east and their lee-side to the west of the quarry. Further west, the ripple-marks terminated by an area covered by wedged and spherical jumbo-desiccation cracks. Again, the jumbo-cracks were filled with centimeter-scale desiccation cracks. At point-J, there was a preserved multi-steps terrace (Fig 18C). Jumbo and small-scale desiccation cracks covered the surface of the preserved-terraces. The ripple marks, desiccation cracks, and terrace-surface cracks were composed of light-olive green silt size sediments. Northwards, the terrace transitioned into two stacks of light-gray paleosols by the northern walls of the creek (Fig 18D). The paleosols were exclusively made of blocky peds and showed no horizonation, redoximorphic features, or root-fossils.

#### Interpretation

The left-lateral strike-slip fault was a local fault, and very likely caused by the rebounding of the modern landscape towards the end of the last great glaciation. The fault exposed two important local morphological features of the Middle Devonian landscape at TCHD, the southwestern edge of a local depression and an abandoned channel that occurred on a distal floodplain. The abandoned channel was mostly covered by the western wall in other parts of the TCHD. Based on the distances between the preserved terraces, it became clear that the channel was more than 100 meters (around 120 meters) wide in this area. Inferred from the low-chroma of siltstone and paleosols as well as the absence of redoximorphic features, it was evident that the channel remained saturated for longer periods. The desiccation cracks, lack of organic materials, silt-sized sediments, and also ripple marks suggested that the pooling of water was shallow and the channel dried out seasonally. The absence of the *Eospermatopteris* fossils was particularly noticeable as the trees were considered to have a preference for the wet environment such as hydromorphic paleosols. The downslope of the local depression was getting steeper by the I-J section as was evident from an increasing thickness of the capping sediments. At a distance of a little over 100 meters, between sections G-H and I-J, the thickness of the paleosol increased from a few centimeters to over 20 cm. Additionally, the wedged-shaped peds, slickensides, and iron-manganese nodules all pointed to the fact that while the moisture-contents of the paleosol fluctuated, the overlying hydromorphic-paleosol received plenty of clay deposits and remained saturated for longer periods. The oxidized micro-pseudoanticlines in the subsurface paleo-vertisol between sections I-J and K-L sections suggested that water percolation wasn’t deep. Cracks at the boundaries of the pseudoanticlines facilitated very limited water percolation.

#### Section K-L

**Fig 19:**
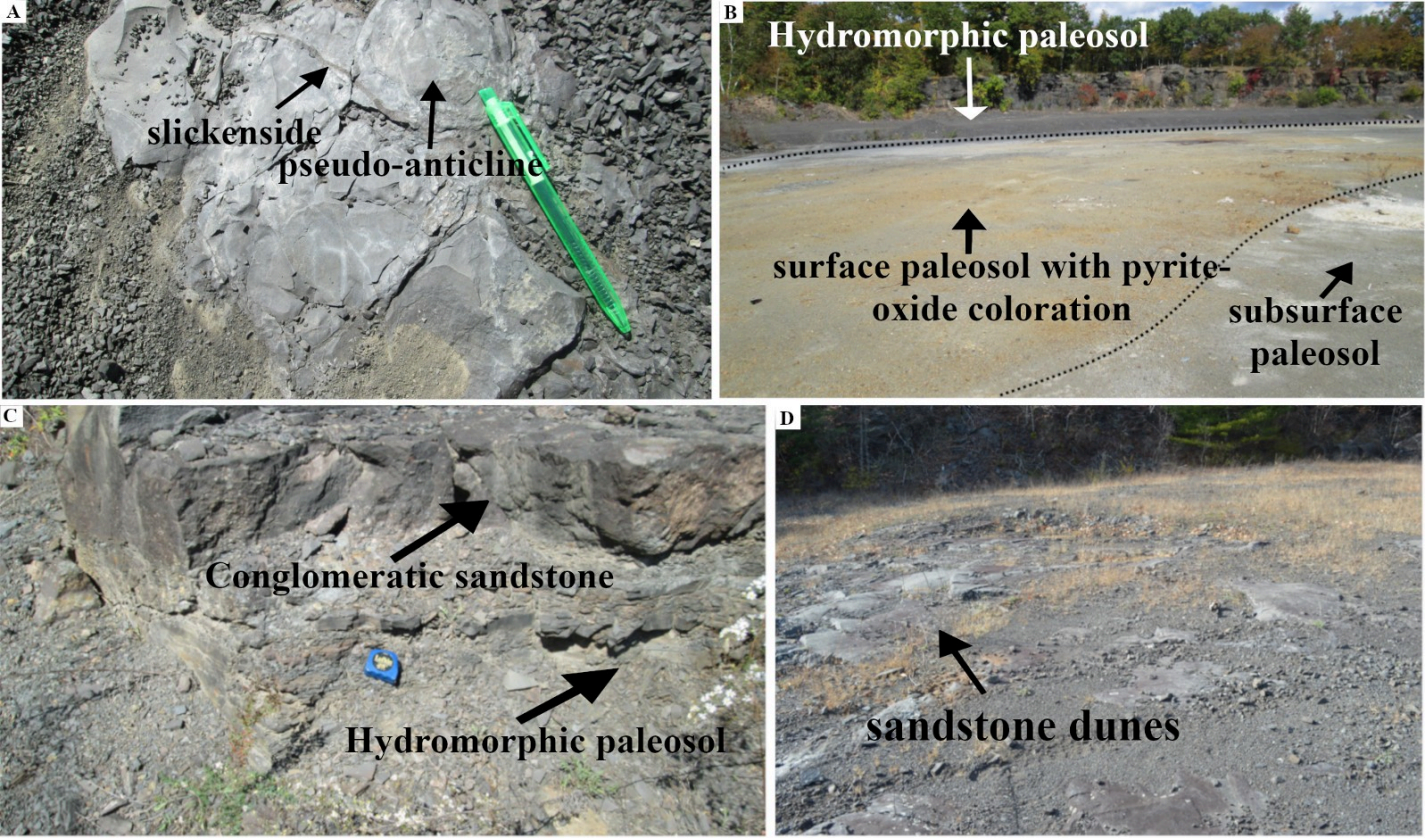
Main features of the paleosols along M-N sections. (A) Pseudoanticlines with slickensided microlows are visible on the surface of the hydromorphic paleosol. The vertic features along the wedged-shaped peds were used as key features to categorize the paleosol as hydromorphic paleosol. (B) The boundary line between hydromorphic paleosol and underlying paleosol. The dotted lines show the transition of the light-olive green peds (subsurface paleosol) into yellowish-brown (surface paleosol) and the overlying light-gray hydromorphic paleosol. (C) Conglomeratic sandstone overlying the light-gray hydromorphic paleosol. The tape measure is at the boundary between the two deposits. (D) The sand-dunes at the N-point of the M-N section. Note the change from conglomeratic sandstone to sand-dunes (the distance is a few meters) in figures (C) and (D).

Three distinct deposits were exposed along the K-L section (Fig 19B). At point-K, more than one-meter thick light-gray paleosol was exposed. It was the topmost paleosol at the K-L section. Wedge-shaped peds and lack of redoximorphic features were the key features of this paleosol. Underlying paleosol was a yellowish-brown paleosol. It was exposed as a result of the quarrying of the overlying light-gray paleosol. The yellowish-brown surface paleosol was composed of silt-sized sediment, blocky peds, pseudo-anticlines, carbonaceous rootlets (Fig 17C), and numerous stem fossils (Fig 17B). The carbonaceous rootlets branched. Some of the stem fossils were curved and their surfaces had a thin outer carbon layer that was identical with the aneurophytale stems reported by Stein from Riverside Quarry at Gilboa, New York [2]. One of the stems was more than 50 cm long, had a broad base, and tapered significantly (from 10 cm to a couple of centimeters). The subsurface paleosol was composed of light-gray peds and large concentrations of the iron-manganese nodules (Fig 17D) that gave the subsurface paleosol the appearance of a “conglomeratic-paleosol”. Towards the point-L, a conglomeratic sandstone overlain over the light gray paleosol. The sandstone had an erosional boundary with underlying paleosol. The conglomeratic sandstone also contained casts of wood logs and fossilized plant materials. Conglomeratic sandstone had pebble-sized clasts with sub to well-rounded shapes.

#### Interpretation

Based on the root and stem fossils, peds and pseudo-anticlines, the yellowish-brown paleosol was correlated with the paleoforest floor at the C-D section. The differences such as silt-sized sediments, yellowish-brown peds, and iron-manganese nodules were because of the variations in the local topography. As we noted before, the C-D section situated on the rise of a local slope, and the paleosol at the K-L section located at a local depression that had poor drainage and remained saturated for a relatively longer duration at a time. Based on the branched rhizoliths, fossilized stems with an outer carbonaceous layer identical to aneurophytales, and another fossil that had a bulged base and high tapering stem, it was possible that all three forms of the Middle Devonian trees including, *Eospermatopteris*, *Archaeopteris*, and anerophytale occupied this part of the local depression. The gray-paleosol with wedge-shaped peds was a hydromorphic paleosol. The deposit hosted the oldest known liverworts fossils [26]. In a 100 meters distance from section I-J, the thickness of the hydromorphic paleosol increased from 20 cm to more than a meter which showed the rapid increase in the angle of downslope or deepening of the local depression at this site. The conglomeratic sandstone was a lag deposit of an active channel that reoccupied the abandoned channel and in the process scoured parts of the underlying hydromorphic paleosol.

#### Section M-N

The M-N section was mostly composed of the light-gray paleosol (Fig 19A), massive conglomeratic sandstone deposits (Fig 19C), and sand dunes (Fig 19D) in the vicinity of the N-point. The surface of the light-gray paleosol was covered with wedged and subspherical-shaped desiccation cracks. Pseudoanticlines that showed slickensides at their boundaries could also be observed on the surface paleosol. The drill core extract at this section showed a light-gray surface horizon with a zone of the iron-manganese nodules (Fig 6G) which transitioned into the organic-rich horizon and then back to the light-gray horizon.

#### Interpretation

The light-gray, wedged-shaped peds and iron-manganese nodules were the key features that were used to categorize the topmost deposits at this section as hydromorphic paleosols and not black shale. The variation of the organic matters in drill core extract at this section recorded the changes in the landscape. The desiccation cracks, pseudoanticlines at the surface paleosol, the increased thickness of the hydromorphic paleosol from G-H to M-N section, and the variations of the organic matter in hydromorphic paleosol suggested a change in the physical features of the local-depression over time. As a result of accretions of clay, silt, and organic matter, the local depression filled up over time. The filling up of the depression changed the drainage from very poorly-drained to a poorly-drained area. The change in the local landscape facilitated the lateral migration of an active channel (Figs 8 and 9) that scoured the western part of the hydromorphic paleosol in the process of reclaiming the abandoned-channel.

### Quarry walls

#### Northwest wall

The sedimentary deposits at the northwestern walls showed the most variations (Fig 8) at the TCHD. The eastern face of the walls that ran along the length of the TCHD didn’t show much variation and shared identical sets of deposits with the southeastern walls. The strike-fault along the I-J cross-section (Fig 2) exposed two additional faces of the northwestern walls. The walls and the gap in between provided a three-dimensional exposure of the sedimentological variations. The abandoned channel deposits, capping sediments as well as lag channel deposits have already been described in the I-J section. The actual walls began with fining-upward sandstone beds. The bottommost sandstone varied from conglomeratic, massive to cross-bedded along the creek. The thickness of the sandstone deposits varied but on average it was around 80 cm thick. The exposed surfaces of the bottommost sandstone deposits showed pyrite-oxidation. The exposed surface of the second set of sandstone showed fossils of plant materials (see S2 Fig via https://doi.org/10.6084/m9.figshare.14558454.v2) including branched stems and around 10 cm thick poorly developed paleosol. A heterolithic deposit of fining upward and trough cross-bedded sandstone interbedded with shale overlaid the second set of sandstone deposits. The shale beds were lensoid. Thickness, colors, and the structure of the shale deposits changed from east to west along the creek. The color of the shale deposits changed from yellowish-brown to light-olive green, light-gray, and dark-gray from east to the west. Likewise, the thickness, dipping angle of the lensoid-shaped deposits, and the fissility of the shale increased from east to west. A set of fining-upward cross-bedded sandstone overlaid the heterolithic deposit. The topmost deposit was composed of poorly-developed paleosol. The paleosol was composed of wedge-shaped peds. The thickness of the paleosol decreased southward and also light-olive green blocky peds replaced wedged-shaped peds. Like topmost paleosol, the overall thickness of the sandstone beds decreased along the length of the quarry from north to the south.

#### Interpretation

The vertical variations of the deposits recorded the behavior of an active channel throughout its lateral migrations. The first sequence of the events was recorded in the form of conglomeratic sandstone, massive sandstone, and cross-bedded sandstone. Conglomeratic sandstone that carried wood logs and plant materials were the lag-gravels from the slumping banks of a migrating channel that reoccupied the abandoned channel. The massive sandstone was channel-fill deposits. Fining-upward trough-cross-bedded sandstone deposits were active channel deposits. The migration of the channel marked the beginning of a depositional environment, and end of the relatively stable floodplain environment that supported the establishment of the forest. The deposition of plant materials and developments of a thin layer of paleosol on the top of the cross-bedded sandstone suggested short-lived stable times in between the lateral migrations of the channel. The second set of depositional events recorded in the form of heterolithic deposits. Flooding and crevasse splays (Fig 10B) in the proximal floodplain deposited the cross-stratified sandstone interbedded with shale. The increase in the size of the shale deposits, their angle, increase in low-chroma, and fissility from east to the west indicated the increasing distance from the channel bank where the grain size decreased with distance. The third and fourth sequences of the depositional events are recorded in the form of cross-bedded sandstone overlaid by the topmost paleosol deposit. The developments of the paleosol suggested a non-depositional environment on a proximal floodplain. The decrease in the thickness of the paleosol from the north towards the south of the quarry indicated that the north was still a low-lying area and hence the possible direction for the paleocurrent. The alternation of deposits in the northwestern walls, the absence of megafossils of plants suggested the relative frequency of lateral migration of the local channel in the absence of vegetative cover (Figs 8–10).

#### North wall

The North wall had identical sets of deposits like the northwest wall with the exception that the cross-bedded sandstone deposits didn’t overlay by paleosols (Fig 8). Other differences were related to the size and geometry of the deposits. For instance, the hydromorphic paleosol that capped forest-floor and likewise sandstone deposits that made the bulk of the quarry walls had the thickest deposits in the north walls. Another difference was the size, geometry, and grain size of the heterolithic deposits relative to the northwest wall. The shale deposits had lensoid geometry but their tapering rate was very low relative to the northwest walls. The color of the shale deposits was yellowish-brown and intercalated with conglomeratic sandstone.

#### Interpretation

While the sequence of the events was similar to the northwest wall (Fig 9A–9F), the differences in the deposits were controlled by the variations in the morphologies of the local landscapes. The northwest wall was situated on an abandoned channel and its bank. In contrast, the north wall was situated in a local depression. That was the reason, the hydromorphic paleosol and the sandstone deposits were thickest in the north walls, and geometry of the shale in the heterolithic deposits did not change much all along the north wall.

#### Southeast wall

The deposits of the southeast walls were identical to the north with the exceptions that the capping paleosol that made the base of the southeast wall showed strong redoximorphic features, and sandstone beds were thinner relative to the north and northwest walls. Additionally the shales of the heterolithic deposits were only a few centimeters (Fig 8). Furthermore, no paleosol was developed on the top of the sandstone deposits.

#### Interpretation

Again, while the sequence of the events was similar to the northwest (Fig 9A–9F) and north walls, the morphology of the local landscape influenced greatly the grain size and thickness of beds. Based on the thick deposits of well-developed vertisols and strong redoximorphic features of capping paleosol, it was evident that the landscape on the southeast wall was situated on relative highlands of a distal floodplain, and was well-drained. After the crevasse splay deposits filled the local depressions, the landscape in the south became more uniform which reflected in the transition of the capping paleosol from well-drained to moderately-drained paleosol, dominance of cross-bedded sandstone, very thin shales, and no paleosol development on the top of the sandstone deposits in the southeast walls and this area remained mostly site of the active channel during the channel’s lateral migrations.

#### Correlations of the north, northwest, and southeast walls

The dip-slip fault in the middle of the TCHD and the strike-slip fault (Figs 2 and 11) at the northwestern wall had caused some relative displacements but because of the short distances between the walls and the lateral traceability of the identical set of the deposits across the quarry walls made it possible to correlate the sedimentary deposits of the north, northwest and southeast walls (Fig 8). Furthermore, several cross-sections from south to the north of the TCHD (Fig 2) made it possible to reconstruct the fossilized landscape and hence helped to understand the controls on the lateral and vertical variations of the deposits. The bottommost deposit in the northeast of the quarry was a paleo-vertisol that hosted the paleoforest floor in the southeast wall and could be correlated to the yellowish-brown paleosol in the north wall that hosted another part of the paleoforest floor. In addition to the paleosols, the preserved local channel and its terraces could be traced from south of the quarry to the northwest walls. The capping sediment that overlaid on the paleo-forest floor was an organic-rich fining-up sandstone that hosted a fallen tree and fish fossils and could be correlated with light-olive-green siltstone by the southeastern wall and light-gray hydromorphic paleosol that hosted liverworts fossils by the north wall and northwest wall. The capping paleosols at the southeast wall and its vertical variations could be correlated with hydromorphic paleosol and its vertical variations at the north wall. Three distinct sequences of the fining upward cross-bedded sandstone, heterolithic deposits and again fining upward cross-bedded sandstone could be found in all three walls. Their thickness and geometry varied between the walls but the sequences of the deposits didn’t change and could be traced in all three walls.

#### Interpretation

TCHD was the site of several cycles of non-depositional and depositional environments, and the local topography played the biggest role in determining the drainage patterns, the distribution of sediments and organisms (Figs 9 and 10). The local landscape was composed of an abandoned channel, its banks, and local depressions at a distal floodplain. The stable landscape and the seasonal pooling of water facilitated the establishment of a mixed forest. The flooding from a crevasse splay changed the seasonal wetland into a long-term waterlogged environment. The floods deposited washed-in young trees, fish, and organic materials which formed a reduced environment. The forest didn’t survive the waterlogged conditions but the reduced environment helped in the preservation of the parts of the forest floor and washed-in fishes and trees. The accretion of clay and silt from the floods changed the local topography by partially filling the local depression. The lateral migration of the channel changed the depositional environment from the distal floodplain to the channel and overbank deposits. The wall deposits recorded three lateral migrations of the channel (Fig 10).

## Discussion

### The shortcomings of the current models to explain fresh evidence

Attempts to model the emergence of complex vascular plant body plans by elementary processes have a long history in botany [27]. In similar fashion, there have been several attempts to link the evolution of plant body plans to particular land environments. Several workers have independently proposed a model of land colonization by earliest forests that suggested a dichotomy of forest ecosystems during the Middle Devonian, where fern-like cladoxylopsid trees [4] or cladoxyl mangals [5] occupied the deltaic environments, and progymnosperm archaeopterids inhabited the floodplains [4, 5]. Similarly, it has been proposed that lowland environments were partitioned at higher taxonomic levels as a result of the origin and radiation of basic body plans which continued till middle-to-late Pensylvannian [6]. Although Retallack associated plants with pedotypes, the associations were based on the vertical exposure of paleosols along road-cuts [5]. The same problem was evident in the suggestion of the partition of lowland ecosystems at higher taxonomic levels in the Late Devonian period [6]. The vertical exposure is useful in studying changes over time. For forest ecosystem studies, the planar exposures of paleosols with in-situ fossils are more suitable. Although no name has been given to this model, this study calls it a size-complexity-gradient hypothesis. From the perspective of this model, the emergence of the partitioning of the forest ecosystems was the result of the constraints imposed by the earliest trees’ body plans. *Archaeopteris* trees had several advanced features that relatively "primitive" *Eospermatopteris* trees lacked. *Eospermatopteris* trees had a central trunk, a body-form common among trees but they also had crowns made of non-leafy, ephemeral branches or fronds, and likewise a bulbous base with an attached mantle of ephemeral non-branched rootlets. The central trunk of these trees was most likely hollow [17] and the number of the vascular bundles increased towards the perimeter of the stem providing mechanical support needed for the upright growth. Trees with these traits were considered adapted for swampy conditions [2, 4, 5]. In contrast, Progymnosperm archaeopterids had central trunks made of wood, a crown made of networks of woody branched stems that bore true leaves, and similarly had roots systems made of woody branching networks that penetrated deep into paleosols. The giant redwoods of California are living evidence for the mechanical support that wood can provide for enormous heights that trees can grow. The partition of terrestrial environments made sense in light of the hypothesis as the advantages that true leaves, wood, and deep penetrating roots provided to the *Archaeopteris* trees were obvious adaptations to unoccupied drier environments beyond the lowland swamps. When Stein reported additional occupants, namely progymnosperm anerophytalean trees and a lycopsid tree in the swampy forests of Middle Devonian lowlands such as Riverside quarry at Gilboa [2], it still made sense, as the lycopsid trees didn’t have true leaves (had leaf-like structures called microphylls that left leaf-base scars on the distal part of the central trunk and crown branches), developed very limited amount of wood (stems developed thin layer of secondary xylem), and had rhizomatic root systems that branched dichotomously (which was very likely a limiting factor in drier environments where drainage pattern was not homogeneous like waterlogged environments). On the other hand, while anerophytalean trees did have woody stems, they adapted a lifestyle of climbing on trees (instead of developing self-supporting upright stems) which were advantageous in any forest with plenty of trees. The complexity of deltaic environments with a forest community like Riverside quarry at Gilboa didn’t challenge the size-complexity-gradient hypothesis. The most recent report from the TCHD where *Archaeopteris*, *Eospermatopteris*, and lycopsid trees occupied an environment that experienced seasonal wetting and drying however could not be explained by the current model. The mismatch between the observed ecosystem and the accepted model posed new questions about the colonization of land environments by the vascular plants. If the earliest forests didn’t evolve from the monospecific forest into forest communities of mixed trees, then, what was the pattern of land colonization by the earliest trees? If trees of different clades had a high tolerance for varied environments, what factors determined the dominance of the *Eospermatopteris* trees in deltaic environments and *Archaeopteris* trees in floodplains? How the variations in the soil-moisture gradients influenced the spatial distribution of the plants with different root morphologies? A new model is needed to answer these questions satisfactorily.

### Environmental variations and processes at the local scale

#### Landscape heterogeneity of a fossilized vernal pool ecosystem

The landscape that made the forest floor at the TCHD was developed on a distal floodplain and broadly composed of three local features, including an abandoned-channel, local depressions, and a woodland at the eastern-bank of the channel. The channel’s features varied from south to the northwest of the quarry following a slope gradient. In the northwest, the channel had multistep terraces, was relatively deeper, showed no redoximorphic features, and had primary sedimentary structures such as ripple marks. In contrast, the southern part of the channel had redoximorphic features, macro-mudslides, pseudoanticlines associated with vertisols, single-step terraces, and well-developed root systems. The significant variations of the local landscape features in a span of a few hundred meters illustrated that the broad environmental classification of the Middle Devonian New York State landscapes between delta and floodplain was an oversimplification. The variations weren’t only limited to the abandoned channel. The local depression also showed variations, although only the southern part of the local depression was exposed which covered the northern half of the quarry. The linear distribution of pseudo-depressions along the southern boundaries of the local depression showed that the wetting and drying processes were more frequent and intense at the edges of the depression. The presence of the micro-pseudoanticlines at the subsurface paleosols between I-J and K-L sections suggested that wetting and drying processes were very slow at the deeper parts. Furthermore, the presence of the redoximorphic micro-pseudoanticlines under hydromorphic paleosols indicated that water didn’t percolate deep and pooled at the surface created a vernal ecosystem. Thin surface paleosol with pyrite-oxide coloration, iron-manganese nodules, and carbonized plant fossils in underlying paleosol along the K-L section supported this conclusion. The wooded area in the eastern bank of the abandoned channel exhibited spatial variations even on a smaller scale. While the wooded area was on a southwest trending gentle slope, it was dotted by the meter scale paleosol mounds. The presence of the mounds were inferred from multiple lines of evidence including the variations of the capping sediments and paleosols, trapped fossils, and the directionality of psuedoanticlines and *Archaeopteris* roots. The local mounds influenced peds-types, sizes, pseudoanticlines, drainage patterns, and redoximorphic features.

The drill core extracts revealed two stacks of well-developed paleo-vertisols with rhizohalos all along the length of the paleosols suggesting a deep water table which made the variations of the landscape even more important for trees at TCHD. The deepwater table meant the surface pooling of the water was very important for the forest establishment and survival, and the variations of landscape features determined the duration and amount of paleosol saturation. The average sediment deposition rate was 0.1 mm year^-1^ [20] during the Middle to Upper Devonian of the Catskill region. Considering that the paleoforest was more than 1.6 meters deep, trees must have flourished for more than 16000 years at this site. Given the estimated old age of the forest, it was very possible that TCHD was not an anomaly but very likely a representative of mature forests of the time that haven’t been preserved or yet to be discovered.

#### Change in the depositional environment by crevasse-splay and preservation of the landscape

The *Eospermatopteris* fossils at the TCHD had a different mode of preservation than the famous Gilboa fossils. The stump-casts were preserved in massive sandstone at Riverside Quarry Gilboa. In comparison, at the TCHD, the preservations were dominantly in the form of trace fossils. The capping sediments varied from fine-sandstone to silts, and hydromorphic paleosol. Although quick burial is key in the preservation of the organisms or their impressions in the forms of fossils, the differences in the sediment grain sizes and modes of preservation strongly suggested that the time, depth, and environment of burial were quite different at both sites. Massive sandstone beds at Riverside quarry that showed almost no lateral changes indicated a vast plain and low lying area. In comparison, the varied sediments at TCHD showed a landscape with diverse features. The diversity of modes of preservation of *Archaeopteris* roots such as root-molds, root-casts, carbonized-roots, rhizohalos also agreed with the diversity of landscape. It also suggested that the initial flooding that quickly buried the forest floor wasn’t massive. The mold of the washed-in young trees and disarticulated carbonized-fish fossils in the capping sediment also illustrated that it was a persistent accumulation of the silt and clay sediments after the initial flooding that preserved the forest floor. The thick deposits of hydromorphic paleosol at M-N section supported the notion that it was the changing of the depositional environment from a seasonal wetland to a permanent wetland that preserved forest floor and the fossils washed-in by the initial flooding. This observation was further supported in the differences of the capping paleosols and modes of fossil preservations between C-D and M-N sections at TCHD. The lower section of the capping paleosol at the point-C showed strong redoximorphic features and tree roots were preserved in the form of the mold trace fossils close to the point-C. In comparison, the lower section of the capping paleosol at the M-N section was composed of organic rich wedge shaped peds, and the root and stem fossils were preserved either in the form of carbonized and partial cast fossils. The casts were carbon rich platy peds that easily broke upon exposure. These differences in duration and depth of burial are an important observation that may explain the rarity of mixed mature forests as well as paleosols formed under those forests in the upland sediments. It’s possible that because of the ideal condition needed for the preservation of the landscapes and organisms, the fossil records were biased towards the low-lying areas, and hence led to the conclusion that *Eospermatopteris* were constrained by their morphologies to the deltaic environments.

#### Change of depositional environment by the lateral shifts of the channel

The correlation of the southeast, north, and northwest walls revealed that basic patterns of the deposits were almost the same (Fig 8). The differences were mainly in thickness of the deposits, and the developments of paleosols. This almost uniform deposit pointed to three key changes that resulted in such a pattern of depositions. a. A crevasse splay deposits that capped the forest also filled up the local depressions with sediments, and leveled off the landscape to a great extent. b. Despite leveling off the local landscape, the northern walls received thicker deposits which indicated a larger scale local sloping from south to north. c. Inferring from the widths of the quarry floor and walls, the active channel that deposited three sets of the cross-bedded sandstone at this location was more than 200 meters wide. The southwestern wall showed the most variation both in sandstone deposits, shales, and paleosol deposits. The conglomeratic, massive sandstone with the pyrite-oxide coloration on their top surfaces, cross-bedded sandstone with plant materials on their top-surface and overlying paleosols all indicated changes from channel to channel-bank deposits as the local channel shifted laterally. The results from the quarry floor and the northwestern wall suggested that the spatial variation in the landscape morphology increased with increasing distance from an active channel (Figs 9 and 10). The observation that landscape diversity increased with distance from the main channels was very important when applied to possible patterns of the land colonization by the earliest forests. The forests that grew in relatively unstable environments such as the ones close to the channel or low lying areas like the young *Archaeopteris* forests reported by the Mintz from the West Saugerties site [4] and also *Eospermatopteris* forests from Riverside Quarry at Gilboa [2] must have had an opportunistic growth.

### Ecological processes at the local scale

#### Spatial distribution of trees at TCHD

Trees belonging to two Middle Devonian tree clades, progymnosperm archaeopterids, and fern-like *Eospermatopteris* distributed along moisture gradients ranged from well-drained, moderately drained to poorly drained surface paleosol (Fig 3). The *Eospermatopteris* of different sizes clustered into two L-shaped clusters around Eosp 1 and Eosp 2 on the south and north of the C-D sections, respectively. Eosp 1 was the largest *Eospermatopteris* tree and occupied the moderately drained area. Eosp 2–4 showed little size variations and occupied a well-drained area. *Eospermatopteris* fossils were also found in poorly drained areas (Fig 3). Tree size didn’t appear to have any influence on their distribution patterns. *Archaeopteris* trees were mostly distributed in an almost linear fashion along the C-D section that showed variations in redox coloration. Like *Eospermatopteris* trees, size didn’t appear to influence the distribution pattern of *Archaeopteris* trees. Another cluster of *Archaeopteris* trees that were less exposed had an almost linear distribution in the vicinity of Eosp 2–4 (Fig 3). As there was only one fossil of the lycopsid tree which was in a moderately drained area, it wasn’t possible to infer the preference of the tree for any particular environment. The finding that size wasn’t an important factor in the distribution patterns of trees at the local scale was an important observation. As arborescence independently evolved in different tree clades, it was evident that natural selection favored size. The size must have had advantages during land colonization. Besides the size, the evolution of different morphologies made it obvious that natural selection also favored various solutions to the environmental stresses of the land environments. It was important to investigate that if size wasn’t the key determining factor in the spatial distribution of the earliest trees at the local scale, then, what other factors influenced the distribution of trees?

The unique linear clustering of trees at the local scale could be a coincidence but we couldn’t be certain that there were no underlying ecological processes that controlled such a distribution. Previous studies have considered drainage patterns as the most important limiting factor [3–5] but as it was noted earlier, the drainage pattern didn’t appear to have influenced the distribution of the trees at the higher taxonomic level, at least not at the TCHD. As an alternative explanation, it could be argued that because it was a seasonal vernal pool environment, drainage patterns were also seasonal, and seasonal environments could have seasonal processes. The seasonality was most likely local because the New York State was at a paleolatitude of 35 S°, and regional seasonality such as moosonal cirulation was not expected for the paleolatitude [28]. Considering the seasonality of the environment at TCHD, we have to see if the trees competed for the areas that remained wet for longer periods, such as poorly-drained areas. Interestingly, although *Eospermatopteris* trees occupied a gradient of poorly to well-drained areas, the majority of them occurred in the well-drained part of the paleo-landscape. If the clustering of trees was considered as an indicator for the competition in trees for certain areas, then no evidence was found that *Eospermatopteris* trees were outcompeted from poorly-drained areas by *Archaeopteris* trees. The occurrence of the *Archaeopteris* trees in the local depression where the subsoil was filled with iron-manganese nodules and likewise, the occurrence of the Arch-6, the youngest of *Archaeopteris* in the abandoned-channel gave an impression that somehow the poorly-drained areas were dominated by the *Archaeopteris* trees. But the absence of any tree in the poorly-drained paleosols in the deeper parts of the abandoned channel such as the northwest part of the quarry (Fig 18A–18D) suggested that trees didn’t compete for the poorly-drained areas, and we have to look for other underlying processes as possible explanations. As all the tree-clades were free-sporing, we can speculate that limitations imposed by the germinations of the spores were the most likely explanation. For germination and likewise protection from desiccation, the spores needed moist soils and shaded areas. Although this study didn’t collect spore data from the forest floor, it was logical to think that the spore density was higher close to the source and the density decreased with increasing distance from the source. Based on this logic we assumed that because of the higher density of the spores near the trees, the number of spores’ germination and seedling survival increased in wooded areas and the areas close to the forests.

It was possible that with increasing distance from the trees, the density of the spores decreased. The low density of spores would also translate into fewer germinations and even fewer surviving seedlings. The viability of spores in modern trees is also very sensitive to relative humidity. For instance, it was found that the mortality rate of spores in some fern species increased sharply above and below 1–25% relative humidity [29]. The linear distribution of the *Eospermatopteris* along south to north (paleocurrent direction) where the largest tree occurred in the south made sense in the light of this hypothesis. The clustering of the *Eospermatopteris* between the C-D and E-F cross-section also supported this hypothesis. Likewise, the almost monospecific forests could also be explained by the limitations of spore viability in areas distant to the forest. It was also important to note the absence of trees in the paleosols developed in the proximity of an abandoned channel (Fig 18D), in hydromorphic capping paleosols that developed in the local depression (Fig 19A–19C) as well as the proximity of the migrating channels (quarry wall deposits, Fig 8). The absence of the vegetation in the hydromorphic paleosols that developed in the proximity of the water bodies suggested that in addition for the right environments, a spore source was also needed.

#### Did Middle Devonian forests of New York State follow the self-thinning rule?

The estimated tree density at the TCHD was around 20 trees per 2000 m^2^ or 200 ha ^-1^. The estimation was based on the uncovered fossils of tree roots, and since parts of TCHD were covered, the tree density could be higher. Mintz and Stein have also plan-mapped fossilized forest floors with in-situ roots from New York State [2, 4]which allowed the calculation of tree density at those fossil sites. The tree densities in the two localities of Gilboa and West Saugerties reported by Mintz were 56 trees per 484 m^2^ or 1153 tree ha^-1^ and 19 trees per 126 m^2^ or 1520 trees ha^-1^. In other words, the TCHD had 5.7 times less tree density than the Gilboa locality and likewise, 7.6 times less tree density than the West Saugerties locality. It is worth mentioning that among modern biomes, deserts and grasslands have the lowest tree density that is around 200 trees ha^-1^, and boreal forests and tundra have the highest tree density that is between 1000–1500 trees ha^-1^. The West Saugerties locality was composed of young *Archaeopteris* trees with a mean stump diameter of 17.2 cm and the Gilboa locality had mixed-aged *Eospermatopteris* trees with a mean stump diameter of 21.2 cm. To compare the sizes of the trees, Arch-3 at the TCHD had 8 main roots and a number of its main roots had diameters around 20 cm and more. Compared to West Saugerties’ young trees, these were both mature and large trees. Similarly, the mean diameter of the *Eospermatopteris* trees was 38 centimeters which were bigger than the 21.2 cm reported from the Gilboa locality [4]. The well-developed deep paleosol with deep penetrating roots also indicated an old age forest at TCHD. It appeared that tree density in the Middle Devonian forests of New York had a relationship with the size of the trees. Although we couldn’t show a relationship between population biomass and density of trees such as those of -3/2 power law of the Yoda’s rule [30] due to the nature of the fossil preservations, however, it was apparent that the tree density of a forest decreased with the increase in the tree size. Were the apparent self-thinning of the forests an outcome of competition? The TCHD provided some clues. In the coupled trees such as Arch-1 and 2 and likewise, Arch-3 and 4 the sizes were contrastingly different. While the size differences could have other reasons, we could not rule out the possibility of competition among close neighbors.

#### Root systems morphologies, sizes, and trees stability

The effect of the tree size on the stability of the *Eospermatopteris* trees was obvious. A washed-in full-size *Eospermatopteris* was preserved in the A-B section area (Fig 12A). The tree’s bulbous base didn’t exceed a few centimeters in diameter. In the same area, close to point-A, there was an in-situ displaced stump-like object. As the possible roots or rootlets were buried, we couldn’t identify if it was a stump, and likewise we could not make an association to any tree clade. Based on the presence of *Eospermatopteris* fossils nearby, it was very likely it was a young *Eospermatopteris* tree. The diameter of the stump-like object was around 15 cm (Fig 12B). If we were correct in our identification, then, young *Eospermatopteris* trees had serious stability issues, as very young ones could be washed out and relatively mature ones could be laterally displaced. *Eospermatopteris* with basal diameters of more than 20 cm in diameters couldn’t be easily displaced laterally but they still could be displaced vertically. Among three neighboring *Eospermatopteris* trees that occupied the same environment, and varied in sizes, two of them showed slickensides at the boundaries of rootlets-paleosol mounds and the host paleosol (Fig 4). The Eosp-2 was one of the largest *Eospermatopteris* trees at this fossil site, and the slickenside at its boundary showed that even the largest *Eospermatopteris* trees had vertical movements during seasonal swell-shrink of the paleosol. The carbon contents of the aboveground part of a 3 meter tall *Pseudosporochnus* tree has been estimated to be 837–1300 g C [31]. This study didn’t find evidence that vertical movements of the trees caused any stability issues, as there was no evidence of fallen trees in the vicinity of the *Eospermatopteris* rootlets-paleosol mounds. *Eospermatopteris* trees stabilized themselves as the mantle of the rootlets emanating from bulbous bases hugged the paleosol and created rootlets-paleosol mounds. The slickensides developed during the swell-shrink of the paleosols as the moisture contents of the paleosols changed. *Archaeopteris* trees, on the other hand, didn’t show any slickensides around the stems, however, minor slickensides were observed along the length of some of the main-roots (Fig 14D). As the trees had branching networks of extensive lateral roots and also deep roots, it was unlikely that such minor slickensides posed any stability issues. The roots circumvented the microhighs of the pseudoanticlines (Fig 13B) and while some roots got pinched between adjacent pseudoanticlines (Fig 14D), there was no evidence of root breakage. The lycopsid tree shared the root morphologies of both *Eospermatopteris* and *Archaeopteris* trees with the difference that root branching was limited to the distal parts of its branched root system. Branching at the distal part of the root system was probably explained by the presence of slickensides at the boundary of its rootlets-paleosol mound. Apparently that root morphology played a very important role in stabilizing trees. Trees with relatively higher branching were likely more stable than trees with low branching and non-branching trees.

#### Trees increased the paleosol heterogeneity

The fossilized forest at TCHD was established on the east bank of an abandoned channel on a distal floodplain. From a limited exposure of paleosols at the northwest of the quarry it became evident that the west bank of the channel was barren. The barren and wooded paleosols that were separated by the local channel provided an opportunity to observe the influence of the tree roots on the paleosol developments. As trees were one of the five soil forming factors, the best approach was to observe the degree of heterogeneity associated with tree roots at the wooded paleosol. As described in the result section, the paleosol that made the forest floor showed remarkable variations from south to the north of the quarry. Peds, redox coloration, pseudoanticlines, and rhizoliths changed from south to north and likewise from east to the west of the forest floor. The blocky peds were frequent in the rooted areas, platy peds formed at the boundaries of the rhizoliths and the paleosol matrix. Prismatic peds were mostly formed at the borders of the local-depression (the area between cross-sections E-F and G-H). As the peds’ boundaries worked as conduits for the movements of the water, clays, and other solubles, the peds’ variations determined the redoximorphic features. Similarly, pseudoanticline sizes varied in the rooted areas (C-D cross-section area) and were mostly decimeters in size (Fig 5). In contrast, the pseudoanticlines in the local depression that formed in subsurface paleosol didn’t show any evidence of root bioturbation and likewise didn’t show many variations, and their diameters measured in centimeters (Fig 17A). The variable micro-topography created by root-paleosol bonding at the local scale had influenced to a great extent the size variations of pseudoanticlines.

On the scale of the quarry floor, the slope gradient was the chief determinant of the local hydrological patterns that were expressed in the form of the paleosol surface redox colorations. Rhizohalos—the redox coloration around the roots- were trace fossils of the root activity in the paleosols. As redox features were used as a proxy for the drainage patterns, in the similar fashion, the rhizohalos could also be taken as a proxy for the hydrological redistribution by roots. The developments of the platy peds and micro-slickensides around the proximal roots were another indication of hydrological redistribution. As most of the water absorptions took place by short-lived terminal roots, the formation of the platy peds and slickensides around long-lived proximal roots showed that these features formed over longer periods. The root size variations from proximal to distal parts not only influenced the water redistribution but also influenced the modes of preservations. The Arch-5 proximal roots were preserved as mold fossils. The distal and terminal roots were mostly preserved in the form of the root-casts, carbonized roots, or rhizohalos. Apparently besides the local slope, the size of the roots also played a role in modes of preservation. In comparison to the heterogeneity of the features that the forest floor showed, the paleosol at the west bank was almost homogeneous. It was made of light-olive green blocky peds (Fig 18D). Similarly, the only feature that the barren part of the eastern bank of the channel -at the northwest part of the quarry- showed was the jumbo-desiccation cracks. The role of the trees in influencing the heterogeneity of the paleosols became more evident when the forest floor was compared with the capping paleosols that covered the same area and landscape features. The overlying hydromorphic paleosols had fewer features than the underlying forest floor. The platy and wedge-shaped peds, pseudoanticlines and slickensides were common features of the capping paleosols south to north of the TCHD. The variations were mostly in the form of the organic matter, fossils, and redoximorphic features which were the products of the variation in the landscape features. In the south of TCHD, the capping paleosol showed strong redoximorphic features and could have hosted some rhizohalos too. In comparison, the capping paleosol at the north was organic rich, hosted liverwort fossils, and lacked any redoximorphic features. The developments of the wedged-shaped peds, liverworts, rootlets, and pseudoanticlines showed that the capping paleosol had a significant amount of time to moderately develop. Similarly, paleosols that were interbedded in the quarry walls and mostly formed on the top of the cross-bedded sandstones were all homogeneous and lacked in-situ root fossils. The reduction of paleosol features from fossilized forest floor to mostly barren landscapes along the active channel (paleosols formed on the top of sandstone deposits and formed quarry walls) showed that the earliest trees increased the heterogeneity of paleosols. If we considered 0.1 mm year^-1^ accretion to the paleosol thickness, both capping paleosols, and the paleosols formed in the quarry walls were more than 1.9 and 1 meter thick, respectively (Fig 8) which translated to more than 19000 and 10000 years, respectively. There was enough time for soil forming factors to develop paleosol features.

#### Land stabilization by an established forest

The frequent fish fossils in between proximal roots of the *Archaeopteris* trees suggested the entrapment of the bedload in a flooding event. The silt size sediments suggested a waning flow that didn’t erode the land during the flooding but carried enough sediment load that buried the forest floor. The experimental studies have shown that vegetation cover significantly improves channel bank stability. For instance, Smith’s work estimated that when roots made 16 to 18 percent of a bank sediment’s volume, the bank’s erosion resistance to erosion enhanced 20,000 times compared to the banks that lacked vegetation [32]. Trees might have influenced the unloading of sediments by creating barriers which in turn helped the preservation of the forest floor and this observation could be taken as direct evidence of the land stabilization by the earliest forests at the local level.

### Ecological processes at the regional scale

A range of forests with different compositions has been reported from the Devonian Period. At TCHD, *Eospermatopteris*, *Archaeopteris*, aneurophytales, and lycopsid trees formed a mature forest in a vernal pool environment. *Archaeopteris* and *Eospermatopteris* trees were the dominant trees of the forests, while the lycopsid and aneurophytale trees dotted the forest. At Riverside Quarry of the Gilboa village, the forests were dominated by *Eospermatopteris* trees that gave them the impression of monospecific forests. In addition to the *Eospermatopteris* trees climbing aneurophytalean progymnosperm and lycopsid trees dotted the forests but no *Archaeopteris* were reported. Similarly, the Middle Devonian West Saugerties forests were composed of exclusively young *Archaeopteris* trees. Monospecific lycopsid forests were reported from China [33] and Norway [34]. Since it was proposed that plants partitioned the lowland at the higher taxonomic levels [9] during the Devonian period, we explore the possible environmental constraints at the higher taxonomic levels. And then, we also explore the role of ecological processes such as plant sizes, flooding, and past experiences of the forests in shaping the variations in the forest compositions.

#### The environmental constraints of *Eospermatopteris* trees

The stump sizes uncovered from the Riverside Quarry at Gilboa varied greatly. The in-situ trace fossils of the stumps were mapped and measured by Stein [2]. It appears that *Eospermatopteris* trees had unequal growths. Young *Eospermatopteris* trees invested heavily in heights. The stem length and diameter of a washed-in young *Eospermatopteris* tree at the TCHD measured 1.5 meters, and a couple of centimeters, respectively (Fig 12A). As trees matured, the basal parts of the stump grew rapidly than the rest of the stump producing prominent bulbous bases, a distinctive feature of the *Eospermatopteris* trees. The basal parts of the stump in old trees could expand up to 4 times more than the rest of the stem. The circumferences of stump casts of *Eospermatopteris* at Gilboa varied between 50–100 centimeters, and their basal parts measured up to 330 cm [17].

The bulbous bases left crater-shaped trace-fossils in the paleosols. The diameter of the central depressions can be taken as an indirect measurement of the diameter of the bulbous bases. In similar manner, if we take the diameter of the central depression as a proxy for the age of the trees then, the size distributions of the in-situ trace-fossils from the riverside quarry at Gilboa provides important information about the demography of the Gilboa forest. We divided broadly the stump sizes into three categories, young (5–20 cm), mature (20–40 cm), and old (>40 cm). The mapped area of the forest was composed of 22% young, 56% mature, and 22% old trees (Table 1). Stump casts have been recovered from multiple horizons [2, 7]. While the occurrence of the stumps at the multiple horizons suggested the regenerations of the *Eospermatopteris* forests after major floodings, the varied sizes of the stumps including large stump sizes indicated stable environments after flooding for prolonged periods of time that allowed the establishments of the multigenerational forests. Mintz interpreted that a stable landscape over a prolonged time needed for establishment of the multigenerational forests at Gilboa [4]. Additionally he interpreted the random distributions of the large diameter trees as the growth of the first generation trees in a homogeneous environment, and the clustering of the young trees as the growth of second-generation in the unoccupied niches. Assuming a stable environment with no environmental stresses, the demography of Gilboa forests can be explained by the development of the *Eospermatopteris* trees. Considering that the forest was established for a prolonged time in a stable environment, the presence of 22% of old trees (Table 1) suggested high mortality among mature trees. The almost equal number of young trees reinforced this hypothesis as young trees occupied the empty spaces created by the death of old trees. Furthermore, despite the clustering of the young trees, the presence of 22% of young trees suggested two possibilities, one high mortality of the young trees, and alternately, the fast growth of young trees. Based on the Berry model, *Eospermatopteris* trees had indeterminate growth [9]. The model divides the trees into three developmental stages, where the basal parts of the trees grew by the upward dichotomy of individual xylem strands which produced the maximum number of strands by the intermediate stages, and the maximum secondary growth of the xylem strands in mature stages. It seems there were no developmental constraints to suggest the high rate of mortality in old trees. As the xylem strands were limited to the perimeter of the stem, and the bulk of the stems were made of pith or were possibly hollow [17], buckling of the tree stems was possible limitations for the old trees. Stein reported a 6-meter long trunk of the *Eospermatopteris* that was 13 cm at top and 47 cm at the base and was radially compressed. As stumps with larger diameters were known from the Gilboa fossil site, Stein suggested that *Eospermatopteris* trees could grow even taller [17]. In general, cladoxylopsids were considered moderate size trees [8]. Some of the stumps recovered from the Gilboa riverside quarry and collected by the New York State Museum showed significant compressions—New York State Museum Collection Number NYSM-13-1 (see S4C Fig via https://doi.org/10.6084/m9.figshare.14908011.v1) and NYSM-6550 (see S4D Fig via https://doi.org/10.6084/m9.figshare.14908011.v1) for instance compressed to an extent that the stem lost its cylindrical shape—and some stumps had their vascular strands bent (see S4A & S4B Fig via https://doi.org/10.6084/m9.figshare.14908011.v1). The visible vertical and horizontal failures of the vascular strands showed that large *Eospermatopteris* trees struggled to support their aboveground parts. The occurrence of the *Eospermatopteris* at the Town of TCHD also showed the preference of *Eospermatopteris* trees for stable environments. The double stacks of well-developed paleosols at the TCHD suggested a stable environment for prolonged periods. Unlike Gilboa, the TCHD didn’t have a uniform environment. It was composed of an abandoned channel, its banks, and a local depression, and water pooled at the depression only seasonally. These two fossil sites suggested that *Eospermatopteris* could easily adopt landscape and environmental variability but environmental stability was the most important limiting factor for establishments of the *Eospermatopteris* trees. The washed-in full-sized young *Eospermatopteris* tree at TCHD also indicated that the young *Eospermatopteris* that have yet to develop rootlets-paleosol mounds could be easily toppled and washed by floods.

**Table 1:** Demographic distributions of the *Eospermatopteris* trees at the Gilboa Riverside Quarry (After Stein et al., 2012).

Young Stumps		Mature Stumps		Old Stumps		Demographic distribution	
Stump ID	Ave. Inner D	Stump ID	Ave. Inner D	Stump ID	Ave. Inner D	demography	%age
AA1b	6	D4 a	22.5	H9 a	40.5	young (5–20 cm)	22%
A1a	8.5	D4 b	23	I9 a	42.5	mature (20–40 cm)	56%
A3a	10	D4 aa	23	I8 b	44	old (>40 cm)	22%
B3b	11.5	C6 a	23.5	I6 b	48.5		
C2a	12	D3 a	24.5	I6 a	50		
C3a	13	E2 a	25	J5 a	50.5		
C5b	14.5	E3 a	25	J7	50.5		
?C5	15.5	ES d	25	K10	55		
D6	18	ES e	26.5	K10	55.5		
D5a	19	E6 a	28.5	K8	60		
		F9 a	30	K6	62		
		F8 a	30.5				
		F4 a	31				
		F2 a	32				
		F1 a	33				
		FO a	34				
		G5 a	34.5				
		G6 a	34.5				
		G7 a	35				
		G9 b	36				
		H9 a	36				
		H6	39				
		H3 a	39.5				
		H6	40				

The inner diameter of the *Eospermatopteris* paleosol-rootlets mounds from Riverside Quarry at Gilboa was used as a proxy for the basal diameters of the *Eospermatopteris* trees. The *Eospermatopteris* trees were broadly categorized into three age groups, young, mature, and old.

#### The environmental constraints of *Archaeopteris* trees

Like other contemporaneous trees of the time, the *Archaeopteris* trees reproduced by spores and likewise adapted to the drier environments by investing in the developments of the secondary vascular systems. The developments of bifacial cambium that produced secondary xylem (wood) and also secondary phloem made it possible for the *Archaeopteris* trees to grow indeterminately, transport photosynthates to longer distances, and resultantly *Archaeopteris* could grow larger and longer than the rest of the earliest trees. Because *Archaeopteris* trees could grow larger and live longer, it was logical to think that in stable environments, *Archaeopteris* would have dominated the forests. But as *Archaeopteris* reproduced by spores, their establishments in drier environments are puzzling. In modern plants, the gametophytes plant bodies that grow from spores are independent of sporophytes, and unlike sporophytes, they do not have protection from desiccation in the form of the cuticles, and likewise, do not have roots to draw water from the subsoil, and therefore are dependent on the surface water for their survival [15]. The survival of the *Archaeopteris* gametophytes and therefore establishment of the first generation *Archaeopteris* forests on barren lands needed a wetter environment. Based on the equal-sized stumps, random distribution, and poor paleosol developments at West Saugerties locality of New York, Mintz suggested that the young *Archaeopteris* trees had an opportunistic growth in an environment that briefly experienced a subaerial exposure [4]. In-situ vertical trunks of archaeopteridalean trees were also reported from the dark paleosol horizon within Plantekløfta Formation, Svalbard, Norway that was interpreted as wet soils [34]. The young forest at the West Saugerties locality was the first-generation stand, and it provided an important clue about the conditions under which *Archaeopteris* forests started. In comparison, to the West Saugerties, the Riverside quarry was stable for longer periods, and the absence of *Archaeopteris* trees among Gilboa trees provided another hint about the environmental preference of the *Archaeopteris* trees. As *Archaeopteris* could grow well in poorly to well-drained paleosols [4], the absence of the *Archaeopteris* trees from Riverside Quarry is puzzling particularly, when progymnosperms such as aneurophytale grew in the Gilboa forests. The morphological differences in the root systems of the *Eospermatopteris* and *Archaeopteris* have been used to explain the preferences of *Archaeopteris* for the upland landscapes [3]. Production of adventitious roots is a morphological adaptation of plants to flooding [4]. The adventitious root systems of the *Eospermatopteris* indicated their higher tolerance to flooding and that might explain the finding of *Eospermatopteris* from multiple horizons at the Riverside Quarry. In contrast, the branched root systems of the *Archaeopteris* from TCHD were mostly composed of shallow roots which suggested their high oxygen demand and poor tolerance for long-term waterlogging. However, the *Archaeopteris* roots have been found in the poorly drained areas of the landscape with some evidence of pyritization which indicated tolerance for short-term waterlogging (Fig 17C). Pyritization in poorly-drained areas of the forest floor at the TCHD was limited to the surface paleo-vertisol which suggested short-term waterlogging. The reduction of sulfates in surface soil is more rapid than in subsurface soils [22]. Low concentrations of hydrogen sulfide increase the tolerance of plants to several stresses such as heat shock, osmotic stress, salt stress, and pathogens but high concentrations of hydrogen sulfide inhibit plant growth [35]. The evidence from the TCHD suggested that *Archaeopteris* trees could not tolerate long-term waterlogging. The bottom sediments of the TCHD were composed of three stacks of paleosols (Fig 8). *Archaeopteris* roots were present in the two stacks of paleo-vertisol but were absent in the hydromorphic capping paleosol. From opportunistic growth of the first-generation forest at West Saugerties locality, regeneration of *Archaeopteris* forest in paleo-vertisol but failure to regenerate in organic-rich hydromorphic paleosol, we get a picture that *Archaeopteris* gametophytes needed wet environment to survive but couldn’t survive swampy environments, and *Archaeopteris* seedlings needed stable environments for prolonged times to grow into mature forests.

#### The environmental constraints of lycopsid trees

The rate of growth of is an important indication of organisms adaptive strategies to their particular environments. The organisms who allocate enormous resources in early maturation and high reproduction have usually a rapid growth rate and short life spans [14]. Such a life strategy is advantageous in disturbed environments. As lycopsid trees made the bulk of paleozoic coal swamps, it was believed that lycopsids had a very short life span [16] as an adaptation to wetland environments. A possible lycopsid tree at the mature forest of TCHD allowed us an opportunity to compare indirectly the growth rate of the trees at higher taxonomic levels. The growth rate of the arborescent lycopods has been somewhat controversial. Some workers favored a high growth rate of lycopsid trees while others challenged the concept [16]. Does the fossil from TCHD provide an independent line of evidence on the rate of growth in lycopsids? The preserved root system of the lycopsid tree at the TCHD shared morphological features with both *Archaeopteris* and *Eospermatopteris* trees. If we take the size of the root systems as a proxy for the age of the trees then, the comparison of the root sizes between lycopsid with *Archaeopteris* and *Eospermatopteris* trees at the TCHD should provide indirect evidence for the growth rate of the lycopsid tree. The lycopsid tree formed a rootlet-paleosol mound identical to the rootlet-paleosol mounds of the *Eospermatopteris* trees. The mound was composed of a central depression formed by the bulbous base of the tree. The average diameter of the central depressions formed by the *Eospermatopteris* trees at the TCHD was around 50 cm in diameter. In comparison, the central depression formed by the lycopsid tree measured 56 cm. If the forest was an old-grown forest as were indicated by the stacks of well-developed paleosols, and likewise by the large root systems of the trees then, the above-average bulbous base of the lycopsid could be interpreted as a higher growth rate than *Eospermatopteris* trees. The dichotomously branching rhizomatous roots of the lycopsid tree were somewhat identical to the *Archaeopteris* branched root systems. Some of the main roots in *Archaeopteris* trees were around 7 meters long, and in comparison, some of the rhizomatous roots of the lycopsid tree measured around 8 meters long. When comparing the length of root systems or the sizes of the bulbous at higher taxonomic levels, we have to keep in mind the contrasting differences in the developments of the root systems. The root systems in lycopsid trees such as Lepidodendrales were shoot-like and probably like shoots were also photosynthetic [36]. Lycopsids had conservative branching [37] and strictly branched dichotomously. The rhizomatous root system of the lycopsid tree at the TCHD mostly remained at the surface of paleosol. Additionally, it has been proposed that the trunks of Lepidodendron trees were monocarpic [10]. The trunks grew rapidly. The axes which branched dichotomously, and had determinate growth appeared later in the development of the trees. From the fossilized root system at the TCHD, we couldn’t determine the development of the root system, however, we could not rule out the possibility of the later appearance of the dichotomously branching rhizomatous root system. The developments of the slickensides at the boundary of the rootlet-paleosol mounds in a relatively poorly drained part of the landscape (Fig 14B) indicated that the tree moved vertically as the paleosol expanded and shrunk by variations of the paleosol moisture during the seasonal wetting and drying. The slickensides also developed at the boundaries of the *Eospermatopteris* rootlets-paleosols mounds but most of the *Archaeopteris* roots systems didn’t develop slickensides. If the rhizomatous roots were comparable in sizes with *Archaeopteris* main-roots, and appeared early in the development of the tree, the tree would have been more stable and slickensides would not have developed. The early appearance of rhizomatous roots would be preserved in the form of trace fossils with deep cuts through the rootlets-paleosol mound. But the trace fossils of the rhizomatous roots in the rootlet-paleosol mound were relatively shallow. The depth of the trace fossils in the rootlet-paleosol mound was not very informative as the bulbous base of the tree could explain the shallow trace fossils. In case, the rhizomatous roots appeared later in the development of the tree, more than 8 meters long rhizomatous roots were suggestive of relatively rapid growth. Alternatively, if the rhizomatous roots appeared early in the development of the tree, the rhizomes still grew longer than most of the main roots of the *Archaeopteris* trees. Berry and Marshall reported from the AF2 locality of Munindalen, Svalbard five vertical external molds of lycopsid trees recognized as *protolepidodendropsis* preserved in sandstone [34]. The stems of the trees were 5.5–8 cm wide, and some of the stems were only 5.5 cm apart. Based on the thin stems and close distance of the trees, it was evident that the fossils were juvenile trees. The bases of the stems extended downward into dark gray paleosol containing 3.5 mm to 5 mm wide subhorizontal roots. The bases of the stumps recovered from the AF3 locality slightly flared. It was suggested that the *protolepidodendropsis* had cormose bases bearing dense small ribbon-like rootlets [34], but considering varying diameters of the roots, roots might have had some developmental stages. While lycopsid trees showed great diversity, the bulbous base morphology was a shared trait between lycopsid trees from Norway and TCHD. The fossils of the in-situ juvenile lycopsid trees suggested that roots appeared early in the development of the lycopsid trees, and the bulbous bases grew as the trees matured. If the rhizomatous roots appeared early in the development of the trees, and bulbous bases grew as the trees matured then, judging from the slickensides developments around the rootlets-paleosol mound, we could infer that rhizomatous rootlets provided less support to the trees. It was likely that their primary function was to absorb water (and possibly photosynthesis as some workers suggested) was an adaptation to a drier environment. The directionality of the rhizomatic roots (Fig 14B) also hinted at the possible adaptation to the drier environment. Most of the rhizomatic roots of the lycopsid tree showed strong directional growth by occurring southwest-west of the rootlet-paleosol mound. As an alternative, we can assume that despite the large size of the tree, the tree was light enough that the swell-shrink of the paleosol displaced the tree vertically. The limited evidence from the TCHD was not conclusive but it hinted at a probable relatively faster growth rate of lycopsid trees than *Eospermatopteris* and *Archaeopteris* trees. One thing, however, was clear that while lycopsids had a faster growth rate than the rest of the tree clades, they didn’t have a short life span, and very likely lived as long as other tree clades.

If we are not mistaken in our observations that lycopsid trees with rhizomatic roots had relatively rapid growth, early maturation, but almost equal longevity to other tree clades then, we need an environmental or developmental explanation for the extremely low numbers of the lycopsid trees at both Gilboa and TCHD. As TCHD had a stable environment, and experienced seasonal droughts, water stress was the main barrier to spore germination and seedling survival. For the low numbers of lycopsid trees in Gilboa forests, we can only speculate that as *Eospermatopteris* had somewhat comparable rapid growth rates and dense populations, the lycopsids were outcompeted by *Eospermatopteris* trees.

In addition to growing to greater heights, the anatomical transformation of the stems was another important difference among the earliest tree clades, where the amount of wood increased from lycopsids to *Eospermatopteris*, and *Archaeopteris* trees, respectively. Despite the differences in the amount of wood production, the lycopsid trees formed the bulk of the coal deposits of the paleozoic era. Exploring the disparity between the amount of wood production and coal formation at the higher taxonomic level sheds light on the interactions of the plants with their environments. TCHD was a mature forest with representations of the major clades of trees that were established in the stable distal floodplain environment for a prolonged period. Very little organic material accumulated on the forest floor or possibly lost during the burial and preservation. Of the standing trees, only the trace fossils of the root systems were preserved in the paleosol. Dense fine roots are preserved only in poorly drained parts of the forest floor. Of the negligible organic materials accumulated on the forest floor, only microscopic and partially decomposed organic material was preserved. In comparison, the capping paleosol that buried the forest was rich in organic matter, possessed liverwort fossils [26] but showed no evidence of the tree roots. As mentioned earlier, the capping paleosols varied vertically in terms of organic contents where the bottom parts had the highest amount of organic matter, and the top parts had the least. The amount of organic matter is reflected in the color variations. The organic-rich parts had dark grey color, and organic impoverished parts had olive-green color. While the capping paleosol didn’t show any evidence of coal formation, but the lack of organic matter in the old-grown forest floor, and in comparison, the high organic matter contents in the capping paleosol where only nonvascular plants such as liverworts grew illustrated the importance of the environmental factors in the accumulation of carbon. This observation to an extent explains why arborescent lycopsids contributed to Paleozoic coals deposits more than any other tree clades. The Gilboa Riverside Quarry also provided an independent line of evidence on the importance of the environment on the formation of coal. While the *Eospermatopteris* dominant forests of Gilboa were established in the poorly drained environment and the forest floor was rich in organic matter, only some of the vascular strands in the stump cast were coalified. The sandy stump casts explained why the coal didn’t form in this environment. Despite the high rate of sedimentation, the intermittent stable and non-depositional environments prevented the accumulation of sufficient organic matter. As previously discussed, the multiple horizons from which *Eospermatopteris* fossils recovered indicated that there were long enough stable environments between two episodes of sediment deposits that allowed the establishment and growth of a mature forest.

#### The environmental constraints of the aneurophytalean trees

Anuerophytalean have been reported from both Gilboa and TCHD [2, 11]. These two forests were established in environments that were stable for prolonged periods of time needed for the development of mature forests. Gilboa locality mostly formed in swampy forests on the lowland areas, and in comparison, TCHD formed a semi-arid woodland that was established on the vernal pool and an abandoned channel. The presence of aneurophytalean trees in both swampy and seasonal vernal pool environments suggested that aneurophytalean trees could grow in a range of environments. No aneurophytalean tree was reported from young *Archaeopteris* forests of West Saugerties locality of New York, and likewise from Devonian lycopsid forests of China and Norway. Although aneurophytalean trees belonged to progymnosperms, had stems up to 15 cm in diameters but had limited secondary tissue production [1], and used other trees for support. Their developmental limitations might explain their absence from young *Archaeopteris* forests at West Saugerties and their presence at the mature forests of Gilboa and TCHD. Because the aneurophytales used other trees for support, the limited investment in the secondary tissues was very likely an adaptation to grow rapidly to access the sunlight. Considering that aneurophytale fronds didn’t possess leaf-like structures [38] and photosynthesized with their green lateral axes, it is very likely that they preferred open canopies created by the death of old trees, and these might explain the clustering of the aneurophytalean trees [1].

#### What was the role of the plant size in patterns of environmental partitioning at the higher taxonomic levels?

The earliest vascular plants such as *Rhynia* had rootless horizontal stems from which 20–25 cm high erect leafless branches grew [14]. The erect stems were photosynthetic, had determinate growth, and could grow densely without overshadowing each other. The rootless stems were dependent on water for their survival and reproduction. While height provided a reproductive advantage, living close to water bodies, the spores did not need to disperse very far from parent plants and it was likely that plants did not have to compete fiercely for height. These details are important when we study the patterns of diversity in early vascular plants. For instance, while *Rhynia* and Horneophyton made monotypic stands in the Rhynie chert deposits, herbaceous lycopods, Aglaophyton were also reported from the deposits [39]. Compared to these herbaceous stands, two monospecific lycopsid forests, one from China [33] and another from Norway [34] were reported. The *Archaeopteris’* remains were also reported from nearby localities to these fossil sites [33, 34]. Lycopsid forest from China had stigamarian rhizomatous roots, and variable density ranging from 8/m^2^ to 38/m^2^ where smaller trees had higher local density. The lycopsid forest of Norway was composed of trees with cormose bases, ribbon-like small roots, and a density of 14/m^2^. The heights of the lycopsids were estimated to be 2–4 m [34]. The size was a key determinant in tree density. The high density of the lycopsid forests suggested that the dichotomous branches didn’t produce large crowns overshadowing neighboring trees. Considering the possible relationships of the branching patterns of stems, and tree density, could the tree sizes also explain the monospecific lycopsid forests? Or developmental or environmental explanations are needed? The mature forests of New York States in comparison were complex [2, 11]. Was it because *Eospermatopteris* and *Archaeopteris* trees had evolved lateral branching, and had indeterminate growth that resulted in crowns that could overshadow neighboring trees? Furthermore, only one lycopsid tree was reported from the wet forest of Gilboa Riverside Quarry [2] and one from the relatively drier forest of TCHD [11]. Both localities had complex Middle Devonian forests. This very low occurrence of the lycopsid trees in the Middle Devonian complex forests may give an impression that lycopsid trees were outcompeted. If it was the case then, only the combination of both developmental and environmental explanations is needed because, in favorable and stable environments, the trees with indeterminate growth and lateral branches could easily grow large and tall enough to overshadow smaller trees.

The case of the aneurophytale trees are fascinating, as unlike other tree groups that might have adapted biomechanical traits that allowed the development of upright stems, and hence could have a better display of photosynthetic and reproductive organs, the aneurophytales trees adapted rhizomatic stems and very likely used other trees to climb [2]. As aneurophytales needed other trees for support, this growth habit suggested an ecological adaptation that required the existence and persistence of complex forests for long enough periods of time that allowed the evolution of such growth habits. While *Archaeopteris* trees had woody stems, *Eospermatopteris* trees also had secondary growth [9] and hence had stems that could tolerate the climbing aneurophytalean trees. The evolution of Anerophytalean trees in the complex forests of Middle Devonian indicated that there was an arms race for sunlight in the earliest forests, and the density of the tree stands, tree sizes, the branching patterns of the tree crowns, and growth rate were key biological factors in determining forest compositions.

#### Adaptation of the earliest trees to flooding and forest regeneration

Both capping sediments, paleosols, and the washed-in fossils suggested that the forest died as a result of flooding. The variations of the hues in the drill-core extract from the hydromorphic paleosol (Fig 6G) indicated that the water level changed over time. There might have been multiple flooding events but because the site was on the distal flooding plain, only the most widespread flooding could have transported and deposited washed-in fishes and young trees. The question is how the flooding resulted in the demise of the forest? Previous studies hinted that the Middle Devonian forests in the New York State were adapted to flooding. For instance, Stein suggested that *the Eospermatopteris* forest at the Riverside Quarry, Gilboa, New York was standing for a relatively limited period and faced frequent disturbances because of its location at the coastal plain [2]. Likewise, Mintz suggested that the young *Archaeopteris* forest at the West Saugerties, New York was the result of an opportunistic growth [4]. If the earliest trees occupied the river banks and the deltaic plains where the flooding was frequent and adapted to the flooding then, what were their adaptive strategies? Modern trees that have adapted to the frequent flooding mature fast as seedlings are prone to getting washed away by flooding. They tend to extend their roots deep into the soil or develop adventitious roots so they can regenerate after burial by the flooding. Also, their seeds require exposed surfaces to germinate which is the key feature of a flooded landscape.

The deep and very well-developed paleosols that formed at the TCHD indicated that the landscape didn’t face frequent flooding and the two stacks of the paleo-vertisols with identical deep rhizohalos suggested the environmental stability was key for the forest regeneration at this site. However, the behaviors of the *Archaeopteris* roots were markedly different in Arch 1–5 that lived on the east bank of the abandoned channel than Arch-6 that grew in the channel, and also the trees that grew in the local depression. Both the abandoned-channel and local-depression had yellowish brown peds (Fig 17B–17D) and the roots were mostly shallow. Arch-6 was mostly composed of main-roots that had a low tendency to branch. As the local depression and the abandoned channel pooled seasonally and remained saturated longer than the rest of the forest floor, it was possible the shallow rooting was their adaptive strategy. The *Archaeopteris* trees also had roots that penetrated deep into the paleosols as were evident from the drill-core extracts (Fig 6A–6D). The *Eospermatopteris* trees in this study had identical root systems like those of Riverside Quarry, Gilboa, New York. The root system was composed of adventitious rootlets that emanated from bulbous tree bases. The root systems suggested that the trees were adapted to flooding but perhaps not to long-term soil saturation. The likely reason that the forest died was root rot from waterlogged conditions that lasted for a very long time as was suggested by the more than 1.6 meters thick light-gray hydromorphic paleosol (Fig 19B and 19C). The lower portion of the capping paleosol along the C-D section showed strong redoximorphic features (see S3 Fig via https://doi.org/10.6084/m9.figshare.14908014.v1) which suggested that local higher ground briefly drained at times, but the forest still failed to regenerate.

In addition to root morphology, the growth strategy of the *Eospermatopteris* also suggested an adaptation to the flooding. The young washed-in tree in the south of the quarry (Fig 12A) showed a slender long stem, small bulbous base, and small crown. The low tapering rate, tall stem, and small bulbous base suggested that the young trees grew rapidly to reach maturity and increased thickness as the tree aged. It was further supported by another tree fossil from the local depression (Fig 17B). The fossil missed the bulbous base and crown but it had a broad base and showed a high tapering rate. In case it was a partially preserved *Eospermatopteris* tree, then the trees tended to invest more in their bulbous base to create more surface area to accommodate their adventitious rootlets as the tree matured.

While the hydromorphic capping paleosol in the north of the quarry lacked root fossils, its equivalent moderately developed vertical capping paleosol showed some potential rhizohalos in the south of the quarry. Bushes or seedlings might have attempted to regenerate in the south of the quarry where the landscape was relatively higher. Despite a long time as suggested by more than a meter thick capping paleosols as well as by the paleosols developed on the top of cross-bedded sandstone deposits (in the northwestern walls), there was no evidence of forest regeneration. The wetlands formed after the burial of the forest at the local depression as well as at the banks of the migrating channel remained barren which suggested that despite their likely adaptation to the short-lived flooding, forest regeneration was a very slow process and a large part of the wetlands in the Middle Devonian New York was not forested.

#### What determined the resilience of the earliest forests, tree traits, or forests’ past experiences?

There is an alternative explanation for the success and failure of forest regeneration after major flooding and that is the past experiences of an ecosystem. Both Gilboa and TCHD had complex forests but there were multiple forest regenerations at Gilboa, and none at the TCHD. Do the differences in the traits of the dominant earliest forest-making trees explain the differences in the resiliency of the two forests? Do the explanations based on the resiliency of the modern ecosystems explain the regeneration differences between the forests more convincingly? In modern forests, it is not the key taxonomic traits that determine the resiliency of an ecosystem but the historical experiences of an ecosystem. An ecosystem that has experienced disturbances in the past is more likely to survive a disturbance than an ecosystem that is established in a stable environment. The capacity of the past experiences of organisms to abiotic changes to influence their present or future biotic responses is called ecological memory [40]. The differences in past experiences of the two Middle Devonian forest ecosystems that were preserved in rock records of the Gilboa village and TCHD in Upstate New York provided some evidence that helped in explaining this question. At Gilboa riverside quarry, the sand stump casts of the *Eospermatopteris* trees found at multiple horizons suggested forest regenerations. The horizons were mostly composed of massive sandstone and very thin paleosol development. The little paleosol developments [2] and bulbous bases that varied between 6 to 62 cm in diameter (Table 1) indicated little time for paleosol development and rapid forest growth. There are five factors that contribute to the formation of the soils including, time, parent material, climate, topography, and organisms [41]. Considering the roles of other paleosol forming factors, the little paleosol development provided independent evidence for the relatively fast growth of the forests in a relatively high depositional environment. The sand-filled casts suggested quick burial by the deposition of high sediment load. Considering all these observations, it became clear that the *Eospermatopteris-*dominated forests at this site adapted to unstable environments, and were able to quickly regenerate, and grew relatively rapidly into mature forests. The forest at TCHD was dominated by *Eospermatopteris* and *Archaeopteris* trees. The drill core extracts revealed two stacks of well-developed paleo-vertisols that had rhizoliths and rhizohalos all along with the depth of the paleosols. The stacks of the paleosols suggested the persistence of a stable environment, and root fossils in both stacks of the paleosols the regeneration of forests. The hydromorphic capping paleosol that at the deepest part was close to two meters deep and buried the forest-floor lacked tree roots. The subsequent sediments that made walls of the quarry also lacked in-situ rootings. The sequence of the paleo-vertisol developments suggested that forest at the TCHD was established in a stable environment and could regenerate in stable environments. But once the depositional environment became unstable, the forests failed to regenerate. This is an important observation for earliest forests because the *Archaeopteris* trees were considered the earliest modern trees with modern traits such as wood, secondary growth, indeterminate growth, lateral branches, and leaves. With this observation, the idea that trees with modern traits were more successful at colonizing the terrestrial environment became questionable. Additionally, as we noticed earlier *Eospermatopteris* trees were well adapted to unstable environments at the Gilboa riverside quarry and could regenerate after major flooding but *Eospermatopteris* at the TCHD could not regenerate after major flooding. This observation provided additional support for the idea that it was not only the traits of the trees that determined their resiliency. The past experiences of an ecosystem was also a determinant factor.

## Conclusion

The New York State had a semi-arid climate during the Middle Devonian and the three earliest vascular plant clades that independently evolved arborescence established forest ecosystems in year-round wetlands of deltaic environments, riverbanks, and also in vernal-pools that experienced seasonal soil saturation. The duration of soil saturation varied from weeks to months at a time. In vernal pool ecosystems, trees didn’t appear to compete for areas that remained wet for prolonged times. Similarly, the water bodies in the terrestrial environments were not always covered with forests as previously thought. Most of the paleosols lacked fossil plants. Lateral shifts of the meandering river channels were the key agent of the change in the depositional environments in fluvial environments which also influenced the duration of the landscape stability. All tree-clades could have opportunistic growths, and clade-dominated forests were likely a common feature of Devonian landscapes in areas that remained stable long enough for the establishment of forests. The vertical exposure of the sediments that either contained transported plant fossils or limited exposures of in-situ plant fossils were likely responsible for the impression that the Devonian landscapes experienced partitioning of the environments by plants at higher taxonomic levels. This study did not find evidence for tree-clades or tree-sizes as determining factors for the distribution of trees along the local environmental gradients. On the other hand, tree sizes and clades determined the forest density. Young forests could have dense growth. The forests went through the process of self-thinning as they matured. Apparently, the resilience of Devonian forest ecosystems depended on the environments they were established in. Forests in flooding-prone areas showed higher levels of resiliency and could regenerate after major flooding. In comparison, forests that were established in stable areas failed to regenerate after a major flooding, most likely because of the change in the depositional environment. The directional growth of surface roots and the deep penetration of the paleosols by the roots was probably an adaptation to the semi-arid condition that not only helped the trees to overcome the water stress during seasonal droughts, tree-stability during the wet season but also enhanced water percolation, and paleosol developments. The microclimates created by the diversity of the root systems and root-soil interactions resulted in heterogeneous surface paleosols at the local levels. The Devonian Period was not just a period of high phenotypic innovations in the vascular plants but also the period of the emergence of complex forest ecosystems, and complex ecosystem processes.
